# Nutrient Removal by Halloysite-Amended Mineral Matrices and Heavy Metal Retention in Rain Gardens Under Dynamic Hydraulic Flow Conditions

**DOI:** 10.3390/ma19163466

**Published:** 2026-08-17

**Authors:** Agnieszka Grela, Justyna Pamuła, Karolina Łach, Maciej Thomas, Damian Grela

**Affiliations:** 1 Faculty of Environmental Engineering and Energy, Cracow University of Technology, Warszawska 24, 31-155 Cracow, Poland; justyna.pamula@pk.edu.pl (J.P.); karolina.lach@pk.edu.pl (K.Ł.); maciej.thomas@pk.edu.pl (M.T.); 2 Faculty of Electrical and Computer Engineering, Cracow University of Technology, Warszawska 24, 31-155 Cracow, Poland; damian.grela@pk.edu.pl

**Keywords:** rain garden, halloysite, dissolved inorganic nitrogen (DIN), soluble reactive phosphorus (SRP), copper, lead, zinc

## Abstract

Rain gardens are widely used for stormwater treatment; however, the performance of alternative sorbent materials under varying rainfall conditions remains insufficiently understood. In this study, the removal of nutrients and heavy metals was evaluated in laboratory-scale rain gardens amended with halloysite of two grain size fractions (1–2 mm and 2–4 mm) under rainfall events lasting 30 and 120 min. Three column systems were tested: a reference column containing dolomite, sand, and gravel (C1), and two halloysite-amended columns (C2 and C3). Synthetic stormwater containing N–NH_4_^+^, N–NO_3_^–^, P–PO_4_^3–^, Cu, Zn, and Pb was applied. Halloysite improved nutrient removal in all rainfall scenarios compared with the reference column. Dissolved inorganic nitrogen (DIN) removal reached 84% in column C3, whereas soluble reactive phosphorus (SRP) removal reached 85% in column C2. Complete removal of Cu, Zn, and Pb was observed in all columns, highlighting the dominant role of dolomite in heavy metal retention. These findings demonstrate the potential of halloysite as a nutrient-removing amendment in rain garden.

## 1. Introduction

Climate change, increasing urbanisation, and progressive catchment surface sealing significantly affect the quantity and quality of stormwater runoff generated in urban areas. Runoff from roads, car parks, rooftops, and other impervious surfaces constitutes a significant source of pollutants discharged into receiving water bodies, including nutrients and heavy metals [1,2,3]. The most frequently detected contaminants include ammonium nitrogen (N–NH_4_^+^), nitrate nitrogen (N–NO_3_^–^), phosphate phosphorus (P–PO_4_^3–^), copper (Cu), zinc (Zn), and lead (Pb), whose presence originates from traffic emissions, the corrosion of building materials, atmospheric deposition, fertilisers, and other anthropogenic activities [4,5,6]. Elevated nutrient concentrations contribute to eutrophication, whereas heavy metals pose an ecotoxicological hazard owing to their persistence, high bioaccumulation potential, and toxicity to aquatic organisms [7,8].

Nature-based solutions (NbS), including rain gardens, bioswales, infiltration basins, and biofiltration systems, are being increasingly implemented as sustainable stormwater management technologies aimed at reducing runoff volume and improving water quality [9,10]. Rain gardens are considered particularly attractive because they combine hydrological, ecological, and landscape functions while supporting decentralised stormwater management in line with sustainable urban drainage strategies [11]. The effectiveness of rain gardens depends largely on the composition and physicochemical properties of the filter media used in the system [12]. Studies on various filter materials for nutrient and metal removal in biofiltration systems have included sand, zeolites, activated carbon, biochar, compost amendments, expanded clay aggregates, diatomite, and mineral sorbents [13,14,15]. Zeolites are well known for their high cation exchange capacity and effectiveness in removing ammonium ions, whereas activated carbon has a high affinity for organic pollutants and selected metals [16]. Halloysite has also been proposed as a low-cost sorbent for stormwater treatment owing to its porous structure and sorption capacity [17]. Nevertheless, these materials have significant limitations. Sand-based materials typically exhibit limited sorption capacity for dissolved nutrients and metals [18]. The use of activated carbon may be associated with relatively high costs and progressive exhaustion of its sorption capacity [16]. Zeolites and diatomite often show selective affinity for specific pollutant groups, necessitating their modification or combined use to achieve the simultaneous removal of nutrients and metals [19]. Furthermore, limited information is available on the operational stability of many sorbents under the variable hydrological conditions characteristic of natural rainfall events.

Notably, heavy metal removal in biofiltration systems may result not only from the presence of sorptive materials such as halloysite, but also from the mineral properties of substrate components routinely used in rain gardens. Dolomite, which is typically used as an alkalising additive and mineral filter material, may play a particularly significant role in this regard. Its ability to immobilise heavy metal ions is associated with the presence of calcium and magnesium carbonate (CaMg(CO_3_)_2_), its high buffering potential, and the possibility of surface adsorption, ion exchange, and coprecipitation of metals as sparingly soluble carbonates (MeCO_3_, Me—metal cation), or hydroxides (Me(OH)_2_) [20,21,22].

Numerous studies have demonstrated the effective removal of Cu(II), Zn(II), and Pb(II) from aqueous solutions using natural dolomite or dolomite-based materials [20,21]. Albadarin et al. [20] reported that metal adsorption onto dolomite proceeds via both chemisorption and surface precipitation associated with an increase in solution pH. Similar findings were reported by Aziz et al. [21], who demonstrated the high effectiveness of dolomite for the removal of Pb(II), Zn(II), and Cu(II) from aqueous solutions.

Halloysite (Al_4_[Si_4_O_10_](OH)_8_∙4H_2_O), a hydrated aluminosilicate clay mineral belonging to the kaolin group, has attracted increasing scientific interest as a potential sorbent for environmental applications [23,24]. Owing to its tubular morphology, relatively large specific surface area, presence of silanol (≡Si–OH) and aluminol (≡Al–OH) functional groups, and ion-exchange capacity, halloysite has favourable adsorption properties for both inorganic and organic pollutants [25,26]. Numerous studies have also demonstrated the effectiveness of halloysite in removing ammonium ions via ion exchange and surface adsorption [10,27]. Leszczyński [10] described effective NH_4_^+^ removal using weathered halloysite, whereas Jing et al. [27] reported an ammonia adsorption capacity (q_e_) of up to 1.66 mg·g^–1^ depending on operational conditions. Enhanced adsorption performance was additionally observed following material modification or conversion into composite structures, including halloysite–hydrogel systems [28] and halloysite-derived zeolites [29]. Halloysite has also shown promising performance in phosphate and heavy metal removal. Phosphate adsorption studies revealed that the adsorption performance depends on material morphology, pH, and surface modification, with iron-modified halloysite nanotubes achieving considerably higher adsorption capacities than unmodified materials do [13,30]. For heavy metals, numerous studies have confirmed the high affinity of halloysite for Pb(II), Cu(II), and Zn(II), primarily through ion exchange, electrostatic adsorption, and inner-sphere complexation mechanisms [14,15,17]. Acid activation, surface functionalisation with amino groups (–NH_2_), and composite formation can significantly increase the adsorption capacity [15,17].

Despite the growing body of literature on the adsorptive properties of halloysite, several significant research gaps remain. Most previous studies were conducted under controlled laboratory conditions with constant hydraulic parameters and in single-contaminant systems [10,13,14,15,17,27,29,30,31]. Therefore, limited information is available on the behaviour of halloysite under the dynamic hydrological conditions characteristic of real stormwater treatment systems, particularly with respect to variable rainfall duration and intensity and preceding dry periods. Furthermore, studies comparing the effects of different grain sizes of the same halloysite material on treatment performance remain scarce. The simultaneous removal of nutrients and heavy metals in integrated stormwater biofiltration systems is also rarely addressed in the literature.

Therefore, the aim of this study was to evaluate the effectiveness of nutrient and heavy metal removal in model rain gardens using halloysite as a filter medium. Two grain-size fractions of the same sorbent were tested under variable rainfall scenarios representing prolonged rainfall, intense rainfall events, and rainfall episodes following dry periods. The specific objectives were to assess the feasibility of using halloysite as a filter medium in rain gardens, to evaluate the effect of halloysite grain size on pollutant removal efficiency, and to assess rain garden performance under different hydrological scenarios. Additionally, the possibility of the simultaneous removal of N–NO_3_^–^ and heavy metals (Cu, Zn, and Pb) was also investigated, and the potential for applying the developed bed configuration in rain garden construction in accordance with engineering guidelines and recommendations [32] was assessed.

The results provide new insight into the applicability of halloysite as an alternative filter medium in stormwater treatment systems operating under realistic and variable environmental conditions. This work may therefore represent important steps in the discussion, design, planning, testing, and evaluation of rain garden performance not only for water retention, microclimate support, and biodiversity enhancement, but also for improved environmental protection.

The novelty of this study lies in the comprehensive evaluation of halloysite as a filter medium amendment for rain gardens operating under dynamic hydraulic conditions representative of real-world stormwater events. In contrast to previous studies conducted predominantly under batch conditions or at constant hydraulic loading, this work investigates the effects of rainfall intensity, event duration, and preceding dry periods on pollutant removal. Furthermore, two grain size fractions of halloysite were systematically compared to determine their influence on nutrient and heavy metal removal efficiency. The study also represents one of the few integrated assessments of the simultaneous removal of nutrients and heavy metals in halloysite-amended rain gardens, and translates the experimental findings into practical recommendations for the design and operation of filter media in natural stormwater treatment systems.

## 2. Materials and Methods

### 2.1. Experimental Setup and Column Design

The study was conducted using three filter columns, whose composition and filling arrangement are presented in Table 1. Each column consisted of a capped pipe from Erson (Kuźnia Raciborska, Poland), with a length of 1000 mm and an internal diameter of 140 mm. The columns were made of epoxy-coated acrylic, which is resistant to mechanical damage and chemically inert with respect to acids, bases, and salts. The leachate outlet was positioned 200 mm above the base, within the dolomite aggregate layer. A detailed description of the column design is provided in previous publications by the authors [33,34].

The columns served as a laboratory-scale model representing containerised rain gardens that can be used for the retention and treatment of stormwater runoff from rooftops. The first column (C1), designated as the reference column, was designed in accordance with the guidelines of the Water Infrastructure Management Authority in Kraków as an example of a rainwater tank intended for urban applications [32]. In the second and third columns (C2 and C3), designated as the halloysite-amended columns, the filter layer was modified by adding halloysite to the sand layer at a mass ratio of 4:1. In both cases, halloysite of different grain sizes was used (1–2 mm in C2 and 2–4 mm in C3). The halloysite used in the study was obtained from the DUNINO Halloysite Mine, operated by “Intermark” (Gliwice, Poland), while the gravel, sand, and dolomite were purchased from a local store.

### 2.2. Composition of Synthetic Stormwater

Before the experiments, the columns were activated with demineralised water (<0.05 µS·cm^–1^), after which each column was subjected to two types of synthetic rainfall applied sequentially and independently: first nutrient rainfall and, after 8 weeks, metal rainfall. The materials used to construct the columns, including the halloysite, were not pre-washed prior to the experiments. In addition, the columns were not flushed or washed between consecutive experiments.

**The nutrient rainfall** contained three compounds commonly found in stormwater: ammonium (NH_4_^+^), nitrate (NO_3_^–^), and phosphate (PO_4_^3−^) ions. The target concentrations of individual ions were NH_4_^+^ = 5.0 mg·L^–1^, NO_3_^−^ = 15.0 mg·L^–1^, and PO_4_^3–^ = 2.0 mg·L^–1^, respectively. All chemicals were purchased from (Chempur, Piekary Śląskie, Poland). The justification for the selected concentrations and the solution preparation procedure are described in Grela et al. [33] and Grela et al. [34]. The mean pH of the rainfall used in Exp. 1 and Exp. 2 was 6.9 ± 0.1. The values presented in Table 2 are expressed as concentrations of ammonium nitrogen (N–NH_4_^+^), nitrate nitrogen (N–NO_3_^−^), and phosphate phosphorus (P–PO_4_^3−^), calculated on the basis of the mass of nitrogen or phosphorus, respectively, contained in the analysed ion. For this reason, the values given in Table 2 are lower than the nominal ion concentrations used during solution preparation. The notation N–NH_4_^+^, N–NO_3_^−^, P–PO_4_^3−^ maintains consistency with the methodology described in Section 2.5.4.

**The metal rainfall** contained three metal ions expected in the runoff from various roofing materials: copper (Cu), zinc (Zn), and lead (Pb). Their concentrations are listed in Table 2. Synthetic rainfall was prepared by dissolving appropriate quantities of metal salts with a purity of at least 99.0%: Cu(NO_3_)_2_∙3H_2_O, Zn(NO_3_)_2_∙6H_2_O, and Pb(NO_3_)_2_ (Chempur, Piekary Śląskie, Poland). Immediately before each simulated rainfall cycle, 500-fold concentrated stock solutions were diluted with deionised water to the working concentrations used in the experiment. The mean pH of the rainfall used in Exp. 3 and Exp. 4 was 5.4 ± 0.1.

The selected metal concentrations (Table 2) were based on literature data. They correspond to high but realistic values that may occur in runoff from metal roofs (copper and galvanised), particularly during the initial phase of a rainfall event or in areas with elevated rainfall acidity and strong anthropogenic pressure [35]. Taneez et al. [36] reported a mean copper concentration of 3.63 mg·L^–1^ in runoff from copper sheet roofs. Galster and Helmreich [37] reported zinc concentrations of approximately 5.75 mg·L^–1^, whereas in highly urbanised areas such as Paris, values reached 7.8 mg·L^–1^. Lead concentrations of approximately 0.628 mg·L^–1^ were reported by Ogburn et al. [38] who attributed the release of lead to its association with zinc in galvanised sheets and pipes, where lead occurs as an impurity in the zinc used during the galvanisation process.

### 2.3. Rainfall Scenarios

The volume of stormwater entering the rain garden was established in accordance with KEGW recommendations [32]. Detailed explanations of the adopted rainfall intensities are provided in Grela et al. [33] and Grela et al. [34].

**120 min rainfall**—total precipitation of 42.9 mm. The rainfall simulation consisted of adding 0.105 L of synthetic rainfall to the columns at 1 min intervals, for a total volume of 12.6 L. This corresponds to less intense, prolonged rainfall with a recurrence period of c = 10 years for the Kraków area.

**30 min rainfall**—total precipitation of 31.6 mm. The rainfall simulation consisted of adding the following volumes of synthetic rainfall to the columns at 5 min intervals: 1.98, 3.75, 1.51, 0.79, 0.67, and 0.59 L, for a total volume of 9.30 L. This corresponds to intense, relatively short-duration rainfall with a recurrence period of c = 10 years for the Kraków area.

The adopted rainfall scenarios were developed on the basis of local design rainfall characteristics for Kraków in accordance with the KEGW guidelines [32] and previous studies by the authors [33,34]. Both the 120 min event with a total precipitation of 42.9 mm and the 30 min event with 31.6 mm correspond to design rainfall events with a recurrence period of c = 10 years, commonly applied in the design of stormwater management facilities. The first scenario represents prolonged rainfall of moderate intensity, whereas the second represents a short-duration high-intensity storm event characteristic of summer convective phenomena observed in Kraków. The use of both scenarios enabled the evaluation of rain garden performance under hydraulic loading conditions representative of those occurring in the local climate.

### 2.4. Experimental Procedure

The prepared filter columns were used to conduct four experiments in which synthetic rainfall was applied as described above.

The first experiment (Exp. 1) involved three 120 min rainfall episodes conducted at 7-day intervals. The second experiment (Exp. 2) also consisted of three 120 min rainfall events, but with 3-day intervals between them. In both cases, nutrient rainfall was applied, enabling the evaluation of dissolved inorganic nitrogen (DIN) and soluble reactive phosphorus (SRP) removal efficiency for each column as a function of filter medium composition and interevent dry period length.

The third (Exp. 3) and fourth (Exp. 4) experiments simulated column performance under metal rainfall. Exp. 3 comprised two 120 min rainfall simulations, whereas Exp. 4 consisted of a single 30 min simulation. In both cases, a 3-day interval was maintained between successive rainfall episodes. These experiments enabled the determination of the removal efficiency of selected heavy metals and nitrate ions.

During each experiment, representative leachate samples (0.1 L) were collected. In Exp. 1, Exp. 2, and Exp. 3, samples were collected at 30, 60, 90, 120, and 150 min after the start of the rainfall simulation. In Exp. 4, samples were collected every 5 min up to 45 min from the start of the experiment. A zero-time sample representing the composition of the synthetic rainfall was also collected at the start of each experiment. Selected water quality parameters were determined in all samples. Additionally, the volume of water draining from the filter columns was measured throughout each experiment.

### 2.5. Analytical Methods

#### 2.5.1. Halloysite Characterisation

Halloysite was characterised using standard instrumental techniques. X-ray diffraction (XRD) analysis was performed using a SmartLab SE diffractometer (Rigaku, Tokyo, Japan) equipped with a HyPix-400 (2D) semiconductor area detector and a Cu LFF X-ray tube. XRD patterns were collected over a 2θ range of 5–80° with a step size of 0.02°. Crystalline phases were identified using the ICDD PDF-5 database [39].

Specific surface area and porosity were determined using a Quantachrome Autosorb iQ–MP (Anton Paar, Graz, Austria). Nitrogen adsorption–desorption isotherms were measured at relative pressures ranging from 1 × 10^–6^ to 0.995. Cells with an internal diameter of 9 mm were used for measurements. The degassing process was carried out in several stages; the applied parameters are presented in Table 3. The results were analysed using ASiQwin v5.21 software.

The specific surface area (S_BET_) was calculated using the Brunauer–Emmett–Teller (BET) method at relative pressures of p/p_0_ = 0.06–0.20. The total pore volume (V_total_) was determined by the single-point method at p/p_0_ = 0.978. The mean pore diameter (D) was calculated according to DA = 4V_total_/S_BET_. Pore size distributions were calculated from the desorption branch of the isotherms using the Barrett–Joyner–Halenda (BJH) model.

Halloysite morphology was analysed using an Apreo 2 S LoVac scanning electron microscope (Thermo Fisher Scientific, Waltham, MA, USA) equipped with EDS UltraDry and Octane Elect detectors (EDAX Ametek GmbH, Weiterstadt, Germany). The samples were sputter-coated with a 2.5 nm gold layer under an argon atmosphere. Analysis was performed under high-vacuum conditions.

#### 2.5.2. pH Determination

The pH was measured using a multifunction laboratory instrument CX-505 (ELMETRON, Zabrze, Poland). The instrument provides automatic temperature compensation over the range of −5 to 70 °C.

#### 2.5.3. Determination of Nutrients and Metals

The nutrient rainfall was analysed for ammonium (NH_4_^+^), nitrate (NO_3_^−^) and phosphate (PO_4_^3−^), whereas the metal rainfall was analysed for Cu, Pb, and Zn. The analytical methods are summarised in Table 4.

#### 2.5.4. Data Analysis

In accordance with the findings reported by Czarnecki et al. [41], nitrite (NO_2_^−^) concentrations in stormwater are typically very low and considered negligible. Accordingly, dissolved inorganic nitrogen (DIN) can be reliably approximated as the sum of ammonium nitrogen (N–NH_4_^+^) and nitrate nitrogen (N–NO_3_^–^) concentrations. The same assumption was adopted in the present study. DIN was calculated according to (1):

DIN = [N–NH_4_^+^] + [N–NO_3_^–^] (1)

For PO_4_^3−^ ions, P–PO_4_^3–^ concentrations were taken to represent soluble reactive phosphorus (SRP). According to Li et al. [42], SRP represents the dissolved phosphorus fraction, which is typically equated with inorganic phosphates.

For Exp. 1 and Exp. 2, the DIN and SRP values were calculated, and their variations over the course of the experiments was analysed. Removal efficiency was determined in accordance with the approach described in the literature [43] according to (2):

(2) $$Removal\;\%=\left(1-\left(\frac{outflow\;concentration}{inflow\;concentration}\right)\right)\cdot 100\%$$

Nutrient loads were calculated following the approach used in bioretention and stormwater research [44], as the sum of the concentration products of and the corresponding outflow volumes at successive time intervals.

Statistical analysis of the results was performed for the mean values of nutrient concentrations by checking the normality of the data distribution using the Shapiro–Wilk test. Depending on the obtained results (*p* ≥ 0.05 normal distribution, *p* < 0.05 non-normal distribution of the measurement data), ANOVA (when the data obtained for each column were normally distributed) or the non-parametric Kruskal–Wallis test (when the data were not normally distributed) was performed to determine statistical significance (*p* < 0.05).

The determination of removal efficiency on the basis of both pollutant concentration and load is essential for a comprehensive assessment of system performance. The concentration-based removal index reflects the degree of change in pollutant concentration between the inflow and the outflow, but does not account for changes in the volume of water flowing through the system. By contrast, the load-based removal index accounts for the total mass of pollutants transported through the system and therefore integrates changes in both concentration and outflow volume [45].

As the analysed columns represent systems with dynamic flow, load reduction may be greater than concentration reduction. This means that even when a small decrease or a temporary increase in the concentration of a given compound is observed in the effluent, the system may still effectively limit the total pollutant load discharged to the receiving water body by reducing the outflow volume. This phenomenon is of particular importance in bioretention systems, because even when negative concentration-based removal efficiency is observed—resulting, for example, from leaching of nutrients from the substrate or mineralisation of organic matter—positive load-based removal efficiency may still be achieved owing to a significant reduction in outflow volume [45].

## 3. Results

### 3.1. Characterisation of the Halloysite Used in the Study

**Figure 1:**
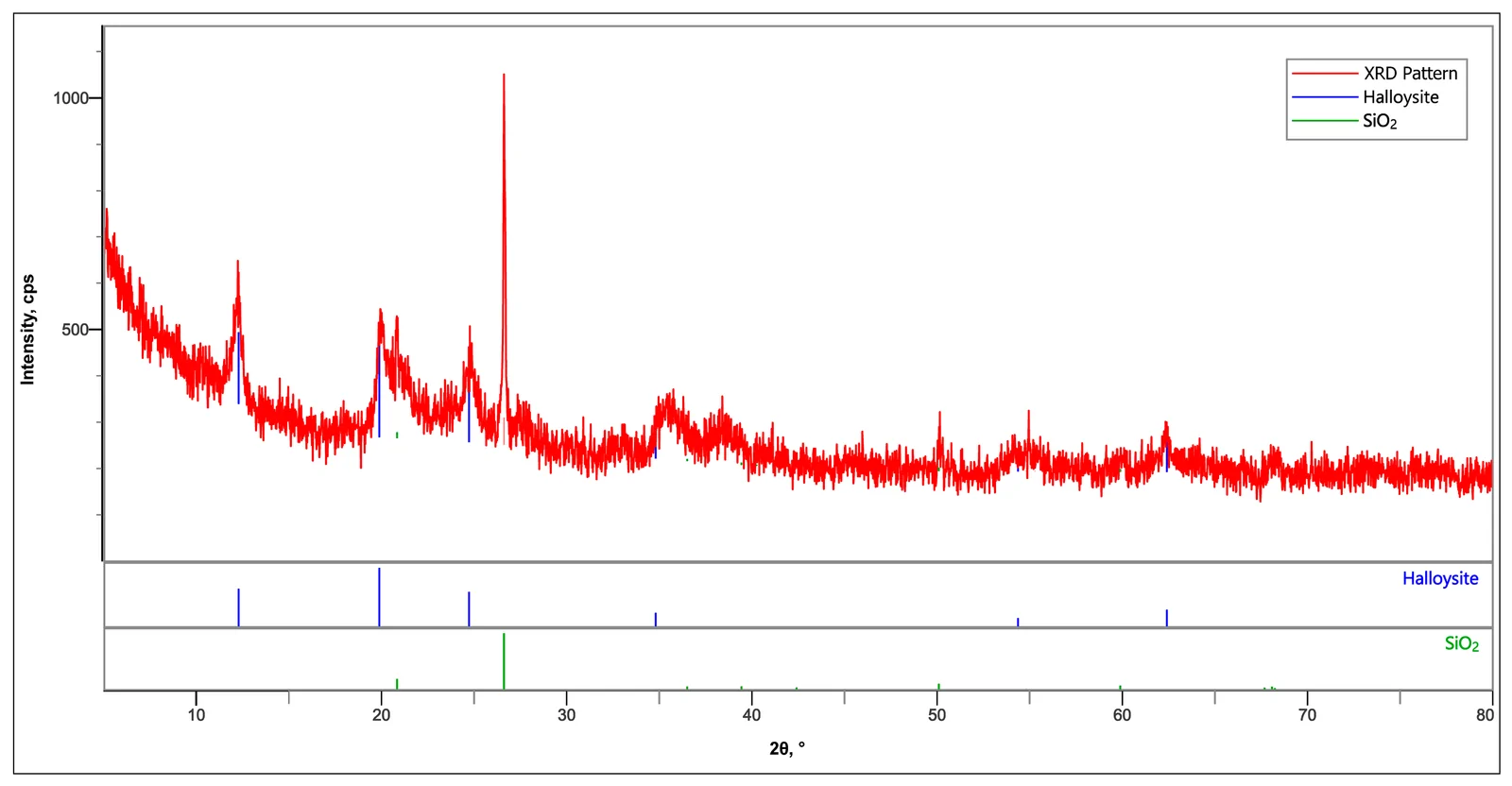
X-ray diffraction pattern of the halloysite sample.

The X-ray diffraction pattern of the analysed sample (Figure 1) confirmed that halloysite was the dominant mineral phase. The diffractogram shows a characteristic broad background with several distinct diffraction peaks, indicating a partially ordered clay structure. The prominent reflection observed at approximately 2θ ≈ 12° is attributed to the (001) basal spacing of hydrated halloysite (001), which is consistent with the literature data for halloysite containing interlayer water molecules. The intensity and shape of this peak suggest a moderate degree of crystallinity and partial structural disorder, which are typical of natural halloysite deposits [23]. Additional reflections visible in the 2θ ranges of approximately 20–25° and 35–40° correspond to higher-order halloysite reflections and may also indicate the presence of associated clay minerals or siliceous phases. The relatively broad and low-intensity peaks suggest poor crystallinity and considerable structural heterogeneity, characteristic of halloysite nanotubes and their aggregated forms [46]. The high background and peak broadening observed in the diffractogram may be associated with the presence of amorphous or poorly crystalline phases, such as amorphous silica or disordered aluminosilicates—a feature frequently reported for halloysite materials formed under natural weathering conditions [47].

The nitrogen adsorption–desorption isotherm obtained for the analysed halloysite sample is characteristic of mesoporous materials. According to the International Union of Pure and Applied Chemistry (IUPAC) classification, the isotherm can be assigned to Type IV, indicating the occurrence of capillary condensation in mesopores. A distinct hysteresis loop is observed, suggesting the presence of slit-shaped pores and interparticle voids commonly associated with aggregated clay minerals. This phenomenon is consistent with the morphological observations from the SEM analysis, which revealed a strongly aggregated structure with considerable interparticle agglomeration. The BET method specific surface area was 55.73 ± 1.1 m^2^·g^–1^ (Table 5), which falls within the typical range reported for natural halloysite materials (20–80 m^2^·g^–1^) [23]. The pore size distribution calculated using the BJH method indicates the dominance of mesopores with a mean diameter of 3.06 nm (Table 5), confirming that adsorption is governed primarily by diffusion within mesoporous structures rather than micropore filling. The total pore volume of 0.22 cm^2^·g^–1^ (Table 5), further confirms the presence of a porous structure of variable density, enhancing the accessibility of the adsorption sites. Such textural properties favour the removal of dissolved pollutants, including ammonium, phosphate, and heavy metal ions, particularly under dynamic flow conditions [46]. Despite its moderate specific surface area, the effective sorption capacity of halloysite can be attributed not only to its textural properties, but also to its surface chemistry and ion exchange capacity, which play key roles in pollutant removal.

**Figure 2:**
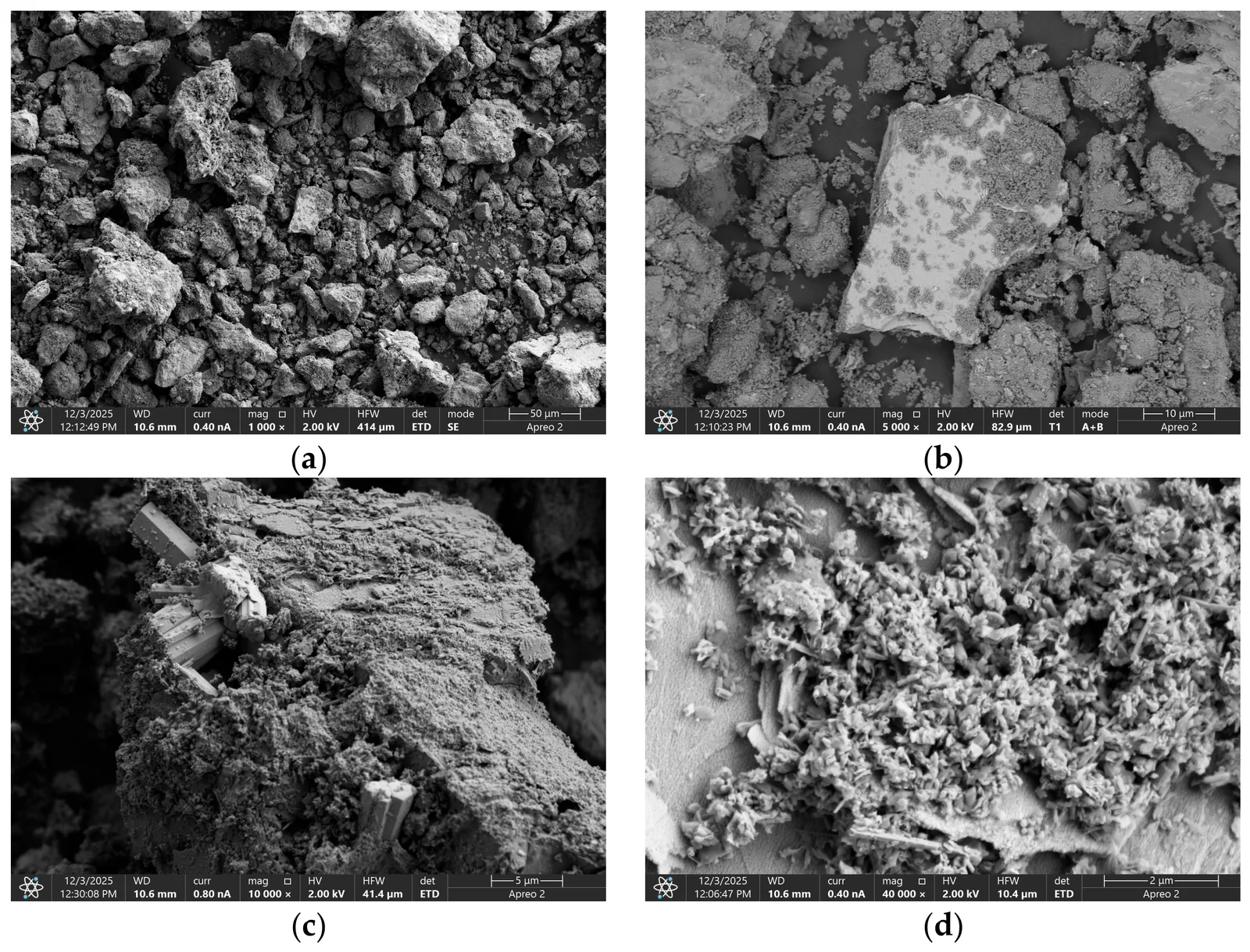
SEM micrographs of halloysite at magnifications of (**a**) 1000×, (**b**) 5000×, (**c**) 10,000×, and (**d**) 40,000×.

The halloysite sample shown in Figure 2a–d is characterised by a highly heterogeneous and aggregated microstructure, which is typical of natural aggregates of this mineral [23,46]. At high magnification, the presence of platy and elongated particles can be attributed to the presence of partially disordered halloysite nanotubes and their aggregates [24,48]. Numerous interparticle spaces and well-developed porosity indicate an enhanced specific surface area, which favours adsorption processes [25]. The presence of fractured structures may increase the number of active sorption sites [47], which is reflected in the high removal efficiency of metal ions and nutrients [11].

SEM–EDS analysis confirmed that the studied material consisted primarily of oxygen, silicon, and aluminium, characteristic of aluminosilicate minerals belonging to the kaolin group. Quantitative EDS analysis revealed elemental contents of 65.8% for O, 15.8% for Si, and 12.6% for Al. Minor contributions of Fe (5.1%) and Ti (0.8%) were also detected. The dominance of Si, Al, and O is consistent with the theoretical chemical composition of halloysite, commonly described as Al_2_Si_2_O_5_(OH)_4_∙nH_2_O.

Halloysite consists of alternating tetrahedral silica and octahedral alumina layers, resulting in the characteristic aluminosilicate composition observed in the EDS spectra [23,46]. The Si/Al atomic ratio obtained in the present study (~1.25) is comparable to values reported for natural halloysite of volcanic and hydrothermal origin, although minor deviations from the ideal stoichiometric ratio may indicate the presence of additional mineral phases or structural heterogeneity [23].

**Figure 3:**
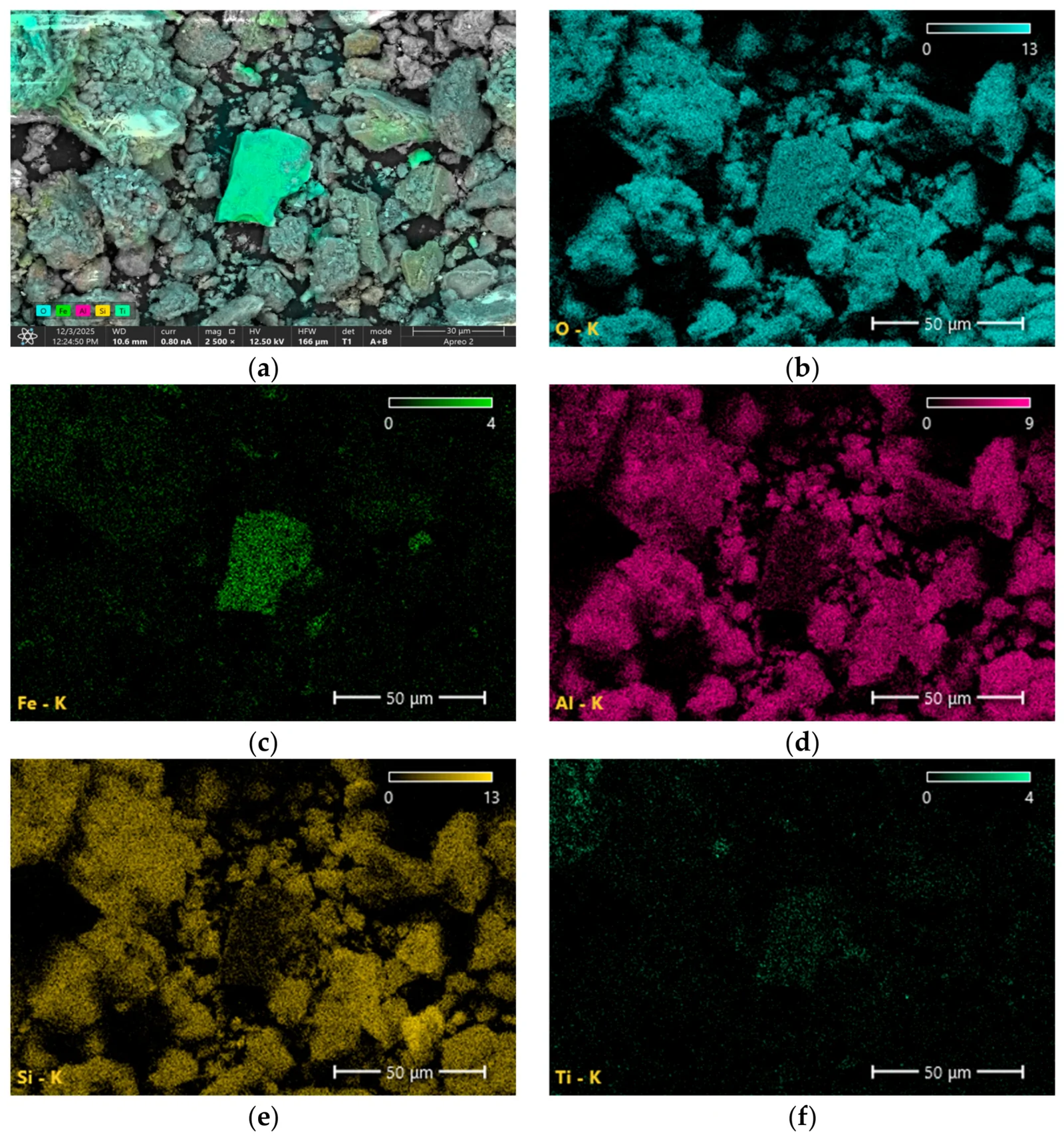
SEM micrograph (**a**) magnified 2500× of the halloysite surface with EDS elemental mapping: (**b**) oxygen, (**c**) iron, (**d**) aluminium, (**e**) silicon, and (**f**) titanium.

Elemental mapping (Figure 3a–f) showed relatively homogeneous spatial distributions of O, Si, and Al within the analysed area, suggesting the uniform occurrence of aluminosilicate phases in the sample. Similar elemental distributions have previously been reported for natural halloysite deposits, in which silicon and aluminium originate from the coupled tetrahedral–octahedral framework of the mineral structure [26,46]. The presence of iron (5.1%) is noteworthy. Natural halloysites often contain iron impurities derived from associated oxides, hydroxides, or mixed clay minerals formed during weathering processes [49]. The relatively high iron content detected in the analysed material may therefore suggest the presence of secondary iron-bearing phases rather than direct incorporation of iron into the halloysite lattice. Iron-rich domains are particularly relevant from the perspective of sorption performance, as iron-bearing sites can significantly increase the affinity of clay minerals for phosphate species and selected heavy metal ions [18].

Titanium was identified in trace quantities (0.8%). The occurrence of Ti in natural aluminosilicates is commonly attributed to accessory minerals such as anatase (TiO_2_), rutile (TiO_2_), or ilmenite (FeTiO_3_), which may cooccur with clay minerals depending on the geological origin of the deposit [50]. Given the low titanium content, its influence on the overall physicochemical properties of the analysed halloysite is considered negligible.

Morphological observations indicate the presence of aggregated particles with irregular geometry and heterogeneous grain size. Distinct, isolated halloysite nanotube structures were not observed, suggesting either strong particle agglomeration or partial co-occurrence of nontubular halloysite forms—a behaviour frequently reported for naturally occurring halloysite samples, whose morphology is strongly dependent on deposit origin, degree of hydration, and sample preparation conditions [46].

From an application perspective, the identified aluminosilicate composition is particularly relevant to absorption processes. Surface hydroxyl groups associated with silanol (≡Si–OH) and aluminol (≡Al–OH) functional groups form active adsorption sites that can participate in ion exchange and surface complexation reactions, as described by (3):

≡ Al − OH + Me^2+^ → ≡ Al − OMe^+^ + H^+^ (3)

These mechanisms are widely recognised as responsible for the adsorption of heavy metals, ammonium ions, and phosphate species by halloysite materials [10,13,18]. The elemental composition obtained in the present study therefore confirms the potential applicability of the investigated material in water and wastewater treatment processes.

### 3.2. Changes in pH

Changes in the pH of nutrient rainfall and metal rainfall after passage through columns C1, C2, and C3 as a function of time (0–150 min and 0–45 min) during Experiments 1–4 are shown in Figure S1a–d in Supplementary Materials, respectively. The initial mean pH of the rainfall was 6.9 ± 0.1 in Exp. 1 and Exp. 2, and 5.4 ± 0.1 in Exp. 3 and Exp. 4.

The experiments revealed that the pH of the rainfall draining from each column differed from the initial values. The influence of column filling on rainfall pH was most pronounced for column C1 (in both Exp. 1–2 and Exp. 3–4), which served as the reference column and contained only gravel, sand, and dolomite aggregates. The maximum pH values in C1 were 9.45, 9.55, 9.37, and 9.47 for Exp. 1, Exp. 2, Exp. 3, and Exp. 4, respectively. Lower pH values were observed in columns C2 and C3, which contained a 20% halloysite amendment. The maximum pH values were 8.61 and 8.21 (C2 and C3, Exp. 1), 8.64 and 8.56 (C2 and C3, Exp. 2), 8.95 and 8.30 (C2 and C3, Exp. 3), and 8.64 and 8.31 (C2 and C3, Exp. 4), respectively. The experiments demonstrated that, under the adopted experimental conditions, the filter-medium composition affected the pH of the column effluent. Alkalisation of the treated rainfall was observed in all the cases, with the effect being slightly attenuated in the halloysite-amended columns.

### 3.3. Nutrient Removal

#### 3.3.1. Nutrient Removal During Prolonged Rainfall Repeated at 7- and 3-Day Intervals

**Figure 4:**
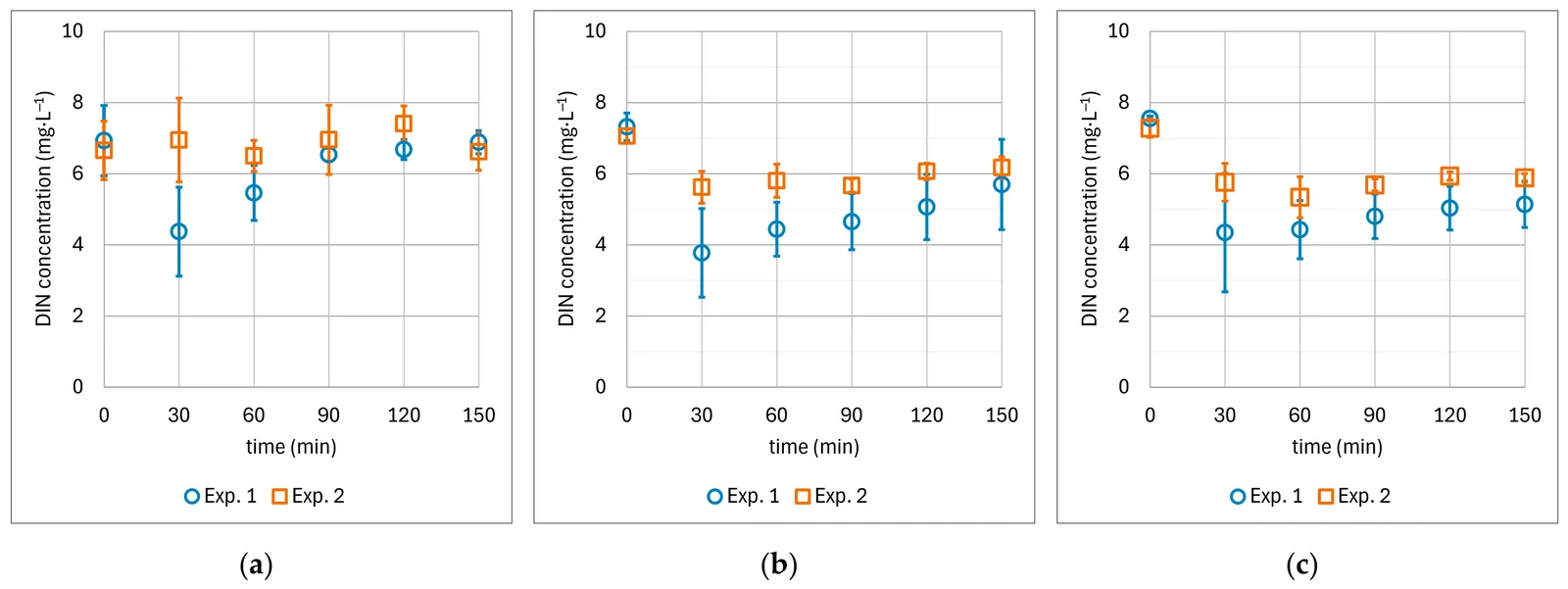
Comparison of mean DIN concentration changes in the three columns: (**a**) C1, (**b**) C2, (**c**) C3, during Exp. 1 and Exp. 2.

The changes in mean DIN concentrations in the three filter columns as a function of rainfall simulation time in Exp. 1 and Exp. 2 are shown in Figure 4a–c, respectively. The error bars represent the standard deviations.

During Exp. 1, in which individual rainfall simulations were conducted at 7-day intervals, a pronounced decrease in the mean DIN concentration was recorded in all columns during the first 30 min of the experiment. These decreases were 37%, 48%, and 42% for C1, C2, and C3, respectively. During the subsequent course of the experiment, a gradual increase in mean DIN concentration was observed up to the end of the simulation at 150 min. In C1, the final concentration was approximately 7 mg·L^–1^, corresponding to only a 1% reduction relative to the initial value. In C2 and C3, the concentration reductions were considerably more pronounced, amounting to 22% and 32%, respectively, with mean final concentrations of approximately 6 mg·L^–1^ and above 5 mg·L^–1^, respectively.

During Exp. 2, in which the interevent interval was reduced by more than half, a change in the character of the DIN concentration dynamics was observed exclusively in C1. In this column, the mean DIN concentration remained at approximately 7 mg·L^–1^, with the greatest change—an increase of 11% relative to the initial value—observed at 120 min. At the end of the experiment, the mean DIN concentration in C1 was 1% lower than the initial value, which is consistent with the results obtained in Exp. 1. In C2 and C3, the concentration trend in Exp. 2 was similar to that observed in Exp. 1: an initial decrease of approximately 20% in both columns, followed by a gradual increase. The final values reached 6.59 mg·L^–1^ and 6.17 mg·L^–1^ in C2 and C3, respectively, corresponding to reductions of 13% and 19% relative to the initial values.

The experiments indicate that, regardless of the rainfall scenario applied, halloysite-amended columns promoted a reduction in mean DIN concentrations compared with those in the reference column. Greater changes in DIN concentration were observed in C2 and C3 than in C1, confirming that lower DIN concentrations were achieved in the halloysite-amended columns. Additionally, halloysite grain size had a modest influence on DIN concentration reduction, with slightly more favourable outcomes observed when a coarser grain size fraction was used.

**Figure 5:**
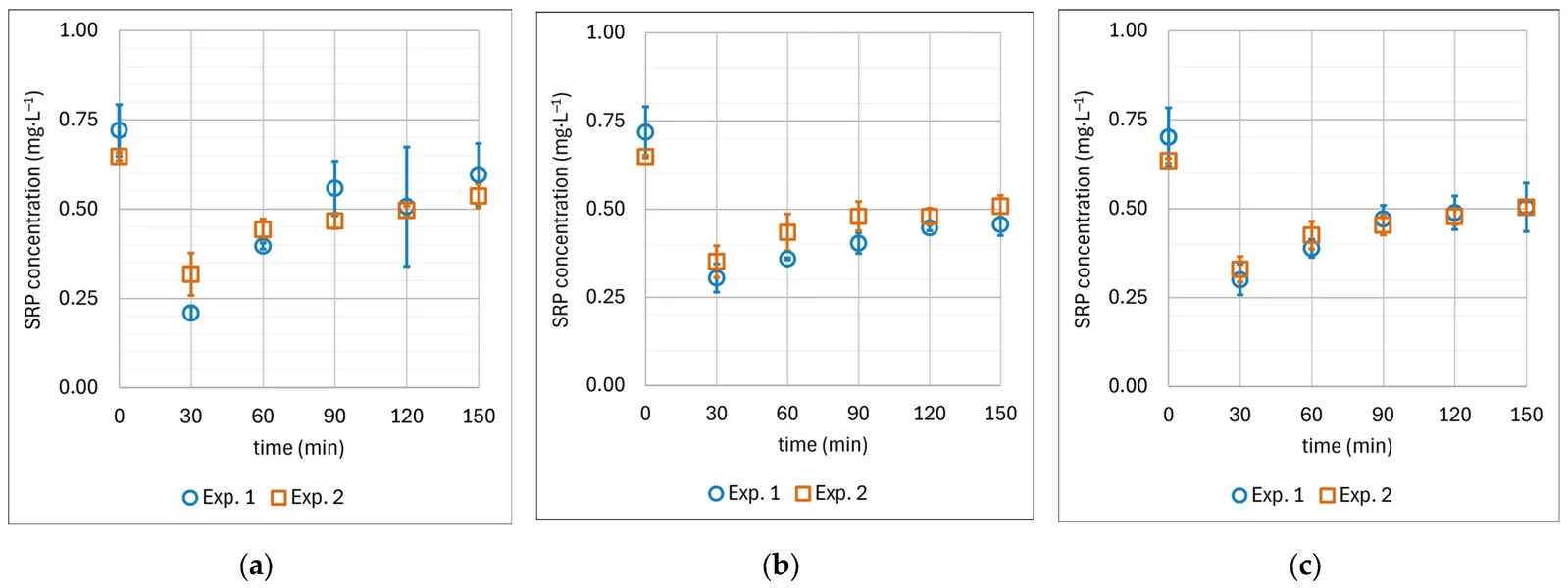
Comparison of mean changes in the SRP concentration in the three columns: (**a**) C1, (**b**) C2, (**c**) C3, during Exp. 1 and Exp. 2.

Changes in the mean SRP concentration in the three columns during Exp. 1 and Exp. 2 as a function of time are presented in Figure 5a–c. The variability of the results is illustrated by error bars representing the standard deviation.

In both experiments, regardless of the column analysed, a pronounced decrease in the mean SRP concentration was recorded during the first 30 min, followed by a gradual increase. Nevertheless, the final concentrations remained lower than the initial concentrations. During Exp. 1, the greatest decrease in the mean SRP concentration after 30 min was observed in C1, which was more than 70% greater than the initial value. In C2 and C3, this decrease was slightly smaller, reaching approximately 60%. At 150 min after the start of the experiment, the lowest mean SRP concentration, 0.46 mg·L^–1^ was recorded in C2. In C3 and C1, these values were 0.50 mg·L^–1^ and 0.60 mg·L^–1^, respectively, corresponding to reductions of 36%, 28%, and 17% relative to the initial values for C2, C3, and C1, respectively. Exp. 2 showed analogous patterns: concentrations decreased by approximately 50% in all columns after 30 min, with final values of approximately 0.5 mg·L^–1^—around 20% below the initial values, with precise reductions of 17%, 21%, and 22% for C1, C3, and C2, respectively.

Compared with the reference column, the halloysite-amended columns resulted in lower SRP concentrations in both experiments. Regardless of the rainfall scenario applied, the greatest reductions in the SRP concentration were recorded in C2. In column C3, the reduction in the SRP concentration was slightly lower but still exceeded that obtained for C1. During Exp. 1, the differences between C2 and C3 were more pronounced than they were in Exp. 2. The results indicate that, for SRP removal, more favourable concentration reductions were achieved when the finer halloysite grain size was used.

Based on the statistical analyses, no statistically significant differences were identified among the three configurations (C1, C2, and C3) with respect to the nitrogen species contributing to DIN (*p* = 0.1345, *p* = 0.0841, *p* = 0.0945, and *p* = 0.4258 for NH_4_^+^ removal in Exp. 1 and Exp. 2 and for NO_3_^–^ removal in Exp. 1 and Exp. 2, respectively) or SRP (*p* = 0.8552 and *p* = 0.9666 for PO_4_^3–^ removal in Exp. 1 and Exp. 2, respectively).

#### 3.3.2. Nutrient Removal on a Load Bases

**Figure 6:**
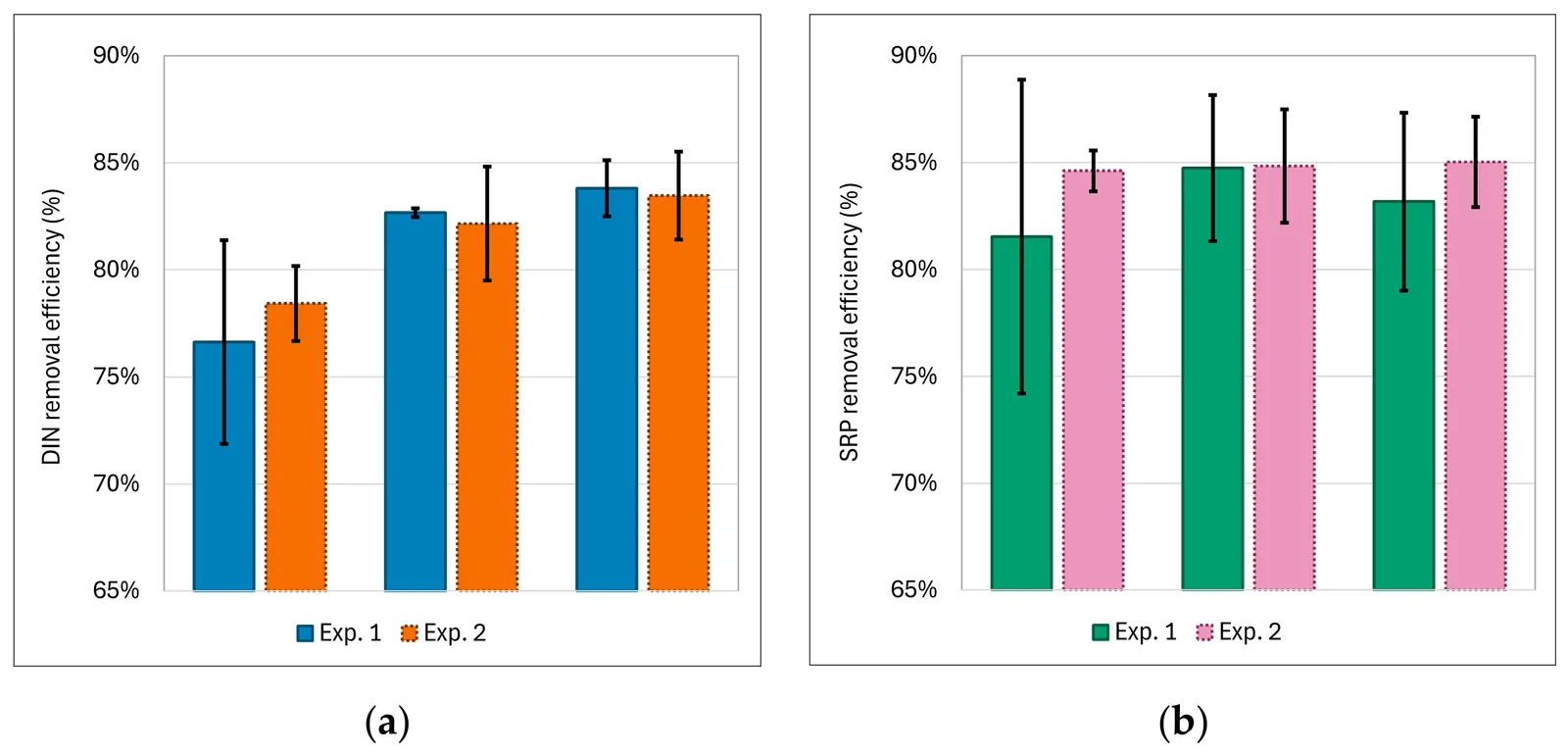
(**a**) DIN and (**b**) SRP mean load removal efficiency during Exp. 1 and Exp. 2.

The performance of each column was also evaluated on the basis of the mean DIN and SRP load removal efficiency during Exp. 1 and Exp. 2, as presented in Figure 6a,b. The error bars represent the standard deviation values.

With respect to the DIN, higher load removal efficiency was recorded in the halloysite-amended columns (C2 and C3), with removal efficiency ranging from 82% to 84% across both experiments. For the reference column (C1), which did not contain a halloysite amendment, the DIN load removal efficiency did not exceed 80%. The differences between C1 and the halloysite-amended columns ranged from 5% to 9%. The results indicate that the halloysite amendment increased the DIN load removal efficiency, whereas the effect of grain size between the two halloysite fractions was minor and practically negligible.

With respect to the SRP, the filter-medium composition had a less pronounced effect on SRP removal than on DIN removal. Nevertheless, compared with C1 (approximately 82%), C2 and C3 showed higher SRP load removal (up to 85%), particularly during Exp. 1. C1 was additionally characterised by greater result variability, as evidenced by higher standard deviation values. During Exp. 2, all columns achieved a similar mean SRP load removal efficiency of 85%, indicating comparable phosphorus removal performance regardless of the column filling composition in this experimental variant.

### 3.4. Heavy Metal Removal After a Dry Period During Prolonged Metal Rainfall (120 min) and a Short, Intense Storm Event (30 min)

Analysis of the results from Experiments 3 and 4 (Table 6) revealed substantial decreases in the final concentrations of Cu, Zn, and Pb in all column configurations, regardless of rainfall duration and filter-layer composition. During both the prolonged metal rainfall applied at 3-day intervals (Exp. 3, 120 min) and the short, intense metal rainfall event (Exp. 4, 30 min), the final concentrations of all the studied metals in the effluent remained below the limits of quantification (<0.05 mg Cu·L^–1^, <0.02 mg Zn·L^–1^, <0.10 mg Pb·L^–1^).

The same result was observed in the reference column (C1), which contained no halloysite but the standard substrate specified by the guidelines [32]. These findings indicate that the dominant component responsible for Cu, Zn, and Pb removal was the dolomite aggregate used in the filter layer, rather than the halloysite amendment.

### 3.5. Nitrate Removal in Metal Rainfall (Exp. 3 and Exp. 4) Compared with Nitrate Removal in Nutrient Rainfall

**Figure 7:**
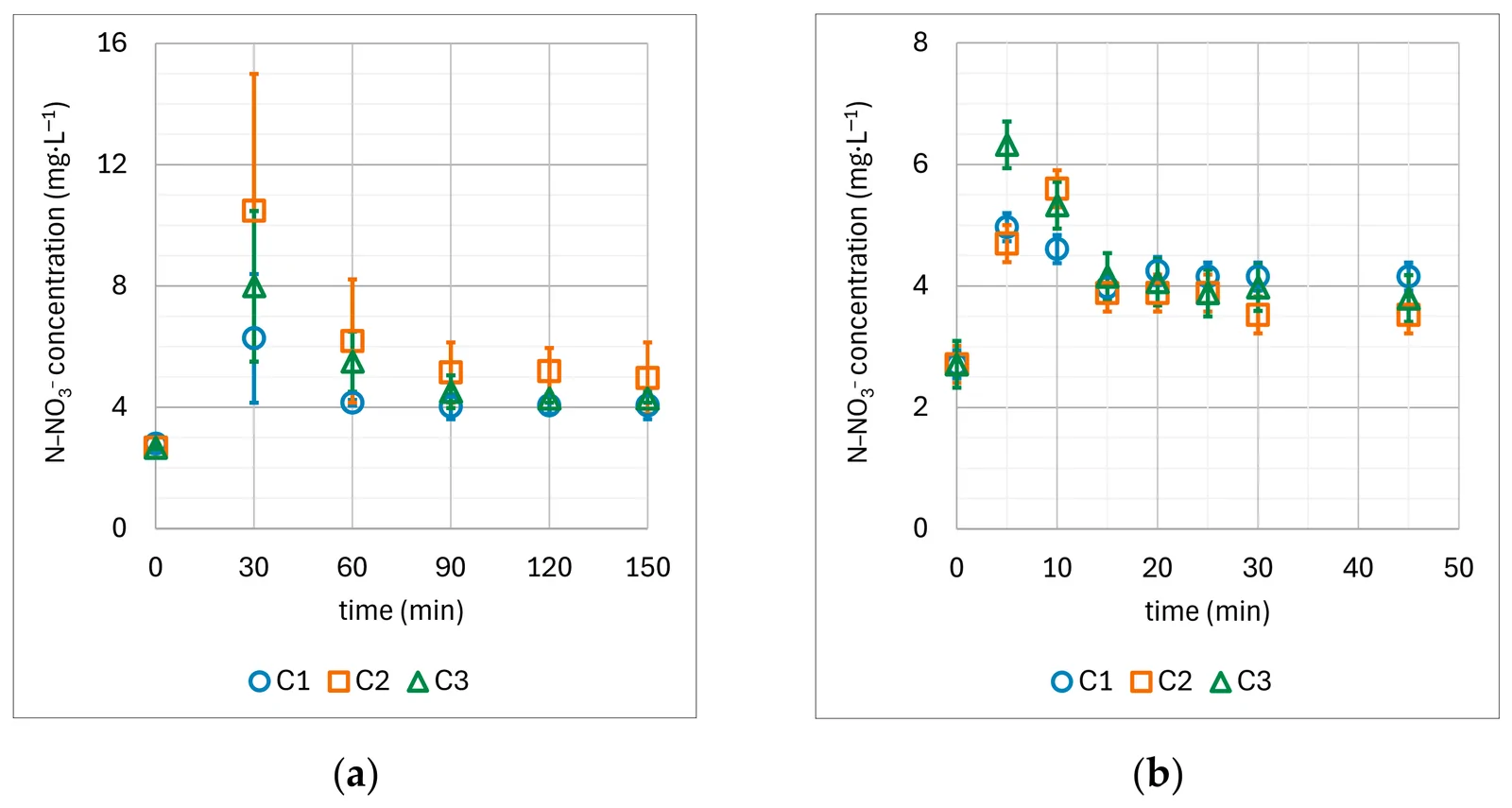
Comparison of mean N–NO_3_^−^ concentration changes in the three columns as a function of time during (**a**) Exp. 3 and (**b**) Exp. 4.

Changes in the mean N–NO_3_^–^ concentrations in the three filter columns during Exp. 3 and Exp. 4 are presented in Figure 7a,b.

During Exp. 3, which simulated prolonged metal rainfall, the greatest changes in N–NO_3_^–^ concentration were observed. In the initial phase, during the first 30 min, a pronounced increase in the N–NO_3_^–^ concentration relative to the initial value was recorded in all columns: more than twofold in C1, approximately threefold in C3, and nearly fourfold in C2. During the subsequent stages, concentrations gradually decreased, reaching similar values in the range of 4–5 mg·L^–1^ at 150 min, but remained higher than the initial values—by approximately 1.5-fold in C1 and nearly twofold in C2.

In Exp. 4, which simulated a short, intense rainfall event, the pattern of N–NO_3_^–^ concentration changes was similar. During the first 5 min, the concentrations increased to approximately twice their initial values, after which a gradual decrease was observed. At the end of the experiment (45 min), the N–NO_3_^–^ concentrations remained higher than the initial values by approximately 30% in C2, 40% in C3, and more than 50% in C1.

**Figure 8:**
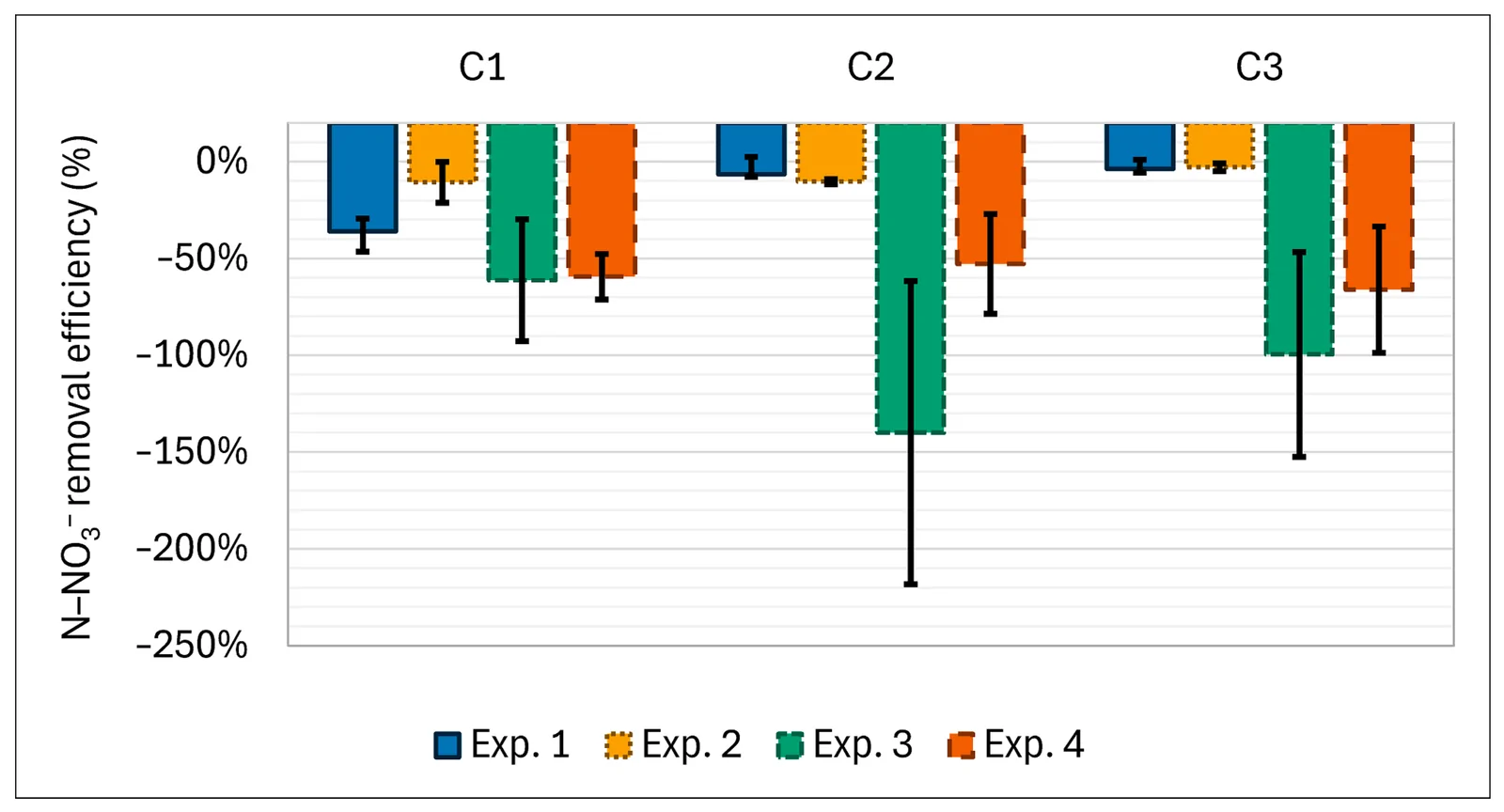
Mean N–NO_3_^–^ removal efficiency across all experiments.

The mean N–NO_3_^–^ removal efficiencies for all the experiments and individual columns are presented in Figure 8. The error bars represent the standard deviation values; negative removal efficiency values indicate an increase in the N–NO_3_^–^ concentration relative to the initial values. Negative values were obtained for all the experimental variants, indicating systematic leaching of N–NO_3_^–^.

In Exp. 1 and Exp. 2, the mean N–NO_3_^–^ removal efficiency was least negative in the halloysite-amended columns (C2 and C3). These values remained slightly below the initial concentrations and did not exceed −10%, whereas the reference column showed greater decreases, reaching −36% in Exp. 1.

In the experiments using metal rainfall (Exp. 3 and Exp. 4), considerably greater increases in the N–NO_3_^–^ concentration were observed. This was particularly pronounced in Exp. 3, where the removal efficiency reached approximately −100% in C2 and −140% in C3, indicating very intense nitrate leaching.

Comparisons of Exp. 3 with Exp. 1 and of Exp. 3 with Exp. 2, conducted at the same rainfall intensity but with different synthetic rainfall compositions, showed the smallest differences were recorded in column C1. In Exp. 3, the N–NO_3_^–^ concentration in C1 increased nearly twofold relative to that in Exp. 1 and by approximately sixfold relative to Exp. 2. In the halloysite-amended columns, considerably greater changes were observed: the increase in N–NO_3_^–^ concentration in Exp. 3 was more than twentyfold greater in C2 and nearly thirtyfold greater in C3 than in Exp. 1, whereas the increase was nearly fifteenfold greater in C2 and more than thirtyfold greater in C3 than in Exp. 2.

A comparison of results for the two metal rainfall events (Exp. 3 and Exp. 4) indicated that the less intense but fourfold longer rainfall event led to greater leaching of N–NO_3_^–^ than the shorter, more intense event did. In Exp. 4, compared with those in Exp. 3, N–NO_3_^–^ concentrations decreased by approximately 1.5-fold in C3 and nearly threefold in C2, whereas in C1 they remained at a comparable level.

## 4. Discussion

The results obtained in the present study, together with an analysis of literature data (Table S1, Supplementary Materials), confirm that halloysite can be used for the effective removal of selected nutrients from water and wastewater; however, process efficiency is strongly dependent on the type of contaminant, the surface properties of the sorbent, and the process conditions. Among the investigated columns, the highest removal was obtained for ammonium ions, followed by phosphate, whereas nitrate removal was least effective.

Natural halloysite exhibits a moderate affinity for NH_4_^+^, which is consistent with previous findings [10,27]. Studies by other authors indicate that the dominant removal mechanisms are ion exchange between NH_4_^+^ and cations present in the mineral structure (Na^+^, K^+^, and Ca^2+^), together with chemisorption at the sorbent surface. Considerably higher adsorption capacities have been reported for modified or composite materials, including halloysite-containing hydrogels and zeolites synthesised from halloysite, indicating that an increased specific surface area and a greater number of active sorption sites substantially improve ammonium ion retention [28,29]. In the present study, halloysite effectively removed N–NH_4_^+^ at the low concentrations characteristic of stormwater and biologically treated wastewater. It should be noted, however, that the flow conditions applied in the experiment limit the contact time between the solution and the material, which may result in lower sorption capacities than those observed in batch experiments [10,27].

For phosphate, the removal efficiency of natural halloysite was considerably lower than for NH_4_^+^ owing to the negative surface charge of the mineral under near-neutral pH conditions and its limited affinity for anions [13,30,31]. The results are consistent with the literature data presented in Table S1 (Supplementary Materials), indicating that natural halloysite has a relatively low phosphate sorption capacity, whereas substantial improvements can be achieved by surface modification with iron or aluminium compounds [30]. The highest phosphate removal efficiencies have been reported for iron oxide-modified halloysite. Almasri et al. [30] demonstrated that the increase in adsorption capacity was associated with Fe–PO_4_ complexes formation and ligand adsorption. It should be noted, however, that the halloysite used in the present study contained 5.1% Fe according to EDS analysis, suggesting that naturally occurring iron-bearing phases may have contributed to phosphate removal, although the form in which iron occurred was not determined. In the investigated columns, the probable phosphate-retention mechanisms were adsorption at aluminol groups and partial retention within the material pores [13,31].

pH crucially affects phosphate adsorption by governing both its speciation and the adsorbent’s surface charge. Under alkaline conditions, HPO_4_^2–^ and PO_4_^3–^ species predominate. When pH exceeds the point of zero charge pH > pH pzc, the adsorbent surface be-comes negatively charged, inducing strong electrostatic repulsion with phosphate anions. Furthermore, competitive adsorption by co-existing OH^–^ ions severely limit the availability of active sites. Therefore, the elevated pH value may have been a limiting factor for the efficiency of phosphate removal [51].

The results confirm that natural halloysite can partially reduce P–PO_4_^3–^ concentrations; however, modified materials may be more suitable for applications requiring high phosphorus removal efficiency.

The lowest removal efficiency was recorded for nitrates. Both the results of the present study and the literature data summarised in Table S1 indicate that natural halloysite has a limited capacity to adsorb NO_3_^–^ [18,52]. According to the literature, this limitation is attributable primarily to the negative charge of the outer surface of halloysite nanotubes and to competition from OH^−^ ions for available sorption sites, particularly at elevated pH [52]. Appropriate surface functionalisation with amino groups may, however, increase the affinity of halloysite for anions by creating positively charged adsorption centres [18]. Only partial N–NO_3_^–^ reduction was observed in the present study, confirming that natural halloysite is not specifically suited to nitrate removal and that improvement of its sorption properties requires surface modification.

Fizir et al. [52] showed that NO_3_^–^ adsorption on halloysite is strongly pH-dependent, reaching a maximum at pH 4 and decreasing as pH increases, which may lead to partial desorption of nitrates. In the present study, the pH became alkaline after only 30 min; this phenomenon may therefore have partially contributed to the observed increase in NO_3_^–^ concentration. Nevertheless, it is unlikely to have been the dominant mechanism because both halloysite and dolomite exhibit a substantially greater affinity for cations than for anions [53].

A more plausible explanation for the observed increase in NO_3_^–^ concentration involves biological processes. Permanent nitrate removal in biofiltration systems occurs mainly through denitrification, which requires anoxic conditions and an available, readily biodegradable organic carbon source [54]. However, aerobic conditions predominated in the investigated columns, and no additional carbon source was supplied, which may have limited denitrification. At the same time, the conditions within the filter bed—including oxygen availability, the presence of NH_4_^+^, pH stabilisation by dolomite, and the developed halloysite surface that could support biofilm formation—may have favoured nitrification [55,56]. As a consequence, a fraction of the NH_4_^+^ may have been biologically oxidised to NO_3_^−^, providing the most plausible explanation for the increase in nitrate concentrations observed during the experiments. Direct competition between Cu^2+^, Zn^2+^, and Pb^2+^ ions and nitrate for the same sorption sites is also unlikely because these ions have opposite charges [57]. Nevertheless, an indirect effect of heavy metals on the surface properties of the filter media and on nitrate mobility within the system cannot be excluded.

Sand, which is the basic filter material in rain gardens, may become sorptively saturated relatively rapidly, in some cases after approximately two years of operation [58]. Sorptive amendments are therefore important for maintaining high treatment efficiency and extending filter-bed service life [58,59,60]. Owing to its low acquisition cost, high chemical stability, and sorption properties, halloysite is a promising filter material for stormwater treatment [61,62].

In conventional rain gardens, phosphate removal occurs through filtration, adsorption, precipitation on naturally occurring Fe and Al oxides, and plant uptake; however, the effectiveness of these processes is limited and strongly dependent on substrate properties [58,63]. The removal of dissolved nitrogen forms is even less predictable, as nitrates may be re-leached from the filter bed, particularly following dry periods [60]. Zhou et al. [63] further demonstrated that many operational rain gardens did not significantly reduce DIN concentrations and that SRP concentrations in the outflow frequently exceeded those in the inflow. Against this background, the systematic decreases in both DIN and SRP concentrations obtained in the present study indicate that halloysite may be an effective filter-medium amendment. Further field studies are nevertheless required to evaluate its long-term performance under real operating conditions.

The improved DIN and SRP removal observed in the halloysite-amended columns indicates that halloysite contributed primarily to nutrient retention, most likely through sorption and ion-exchange processes. Although the statistical analysis did not identify significant differences among the studied configurations in DIN or SRP removal (*p* > 0.05), the halloysite-amended columns showed favourable descriptive trends in nutrient retention. The lack of statistical significance may reflect the limited number of replicates, the relatively small differences among the variants, and the variability characteristic of dynamic-flow experiments. From a practical perspective, the observed differences may be relevant to filter design, but they require confirmation in studies with a larger number of replicates and under field conditions.

The present study showed near-complete removal of Cu, Zn, and Pb even in the reference column (C1), which contained no halloysite. This result indicates that dolomite in the filter layer played the dominant role in heavy metal retention. The halloysite amendment did not produce a measurable increase in metal removal efficiency, and the use of two grain-size fractions (1–2 mm and 2–4 mm) did not significantly affect process performance. This is likely attributable to the very high effectiveness of dolomite and the relatively low initial metal concentrations (Cu ≈ 3 mg·L^–1^, Zn ≈ 6 mg·L^–1^, and Pb ≈ 1 mg·L^–1^), at which the number of available sorption sites substantially exceeded the quantity of incoming contaminants.

The observed differences between the two halloysite grain-size fractions can be explained by the interplay among specific surface area, mass transfer, and the hydraulic properties of the filter medium. The finer fraction (1–2 mm) provides a larger external surface area and a higher density of accessible adsorption sites, resulting in shorter diffusion paths and faster adsorption kinetics. These characteristics particularly favour phosphate removal, which, according to the literature, occurs mainly through surface complexation at aluminol and silanol groups. In contrast, the coarser fraction (2–4 mm) forms a more porous filter matrix with lower hydraulic resistance, facilitating water transport through the material and intraparticle diffusion. Consequently, the slightly higher DIN removal observed for the coarser material may be associated with more efficient ion-exchange processes and longer effective contact within the pore network. Similar relationships among particle size, adsorption kinetics, and diffusion limitations have been reported for halloysite and other aluminosilicate sorbents [10,23,46]. The effect of halloysite grain size should therefore be interpreted in relation to both the sorption and hydraulic properties of the filter bed. Although separate BET pore-size-distribution measurements were not performed for the 1–2 mm and 2–4 mm fractions, both fractions were obtained from the same halloysite material and were therefore expected to have comparable internal textural properties. Overall, the results indicate that the grain-size effect was pollutant-dependent and limited under the experimental conditions.

It should also be considered that the columns contained a dolomite layer, which may release Ca^2+^ and Mg^2+^ during dissolution and thereby influence phosphorus immobilisation and other geochemical processes [64,65]. These ions were not analysed in the present experiments; therefore, their contribution to nutrient removal could not be assessed.

The literature review presented in Table S2 (Supplementary Materials) confirms that natural halloysite has a high affinity for heavy metals, particularly Pb(II) and Cu(II), and a lower affinity for Zn(II) [14,15,16,17,66,67,68,69]. Sorption mechanisms include primarily complexation of metal ions with silanol and aluminol groups, chemisorption, and, to a lesser extent, ion exchange [14,15,66]. Numerous studies have also demonstrated that chemical or thermal activation and composite formation substantially increase the sorption capacity of halloysite by increasing its specific surface area and the number of active adsorption sites [14,15,16,17,69].

Under the present experimental conditions, however, the metal-sorption capacity of halloysite was probably masked by the presence of dolomite. The high heavy metal removal observed in the reference column indicates that halloysite had only a supplementary role, whereas dolomite was the principal material responsible for metal immobilisation. The findings therefore indicate that, in the investigated filter system, halloysite primarily improved nutrient removal, whereas dolomite provided effective heavy metal retention. The two materials thus performed complementary functions and increased the overall stormwater treatment efficiency.

The sorption properties of dolomite towards heavy metals are well documented [20,21]. Cu^2+^, Zn^2+^, and Pb^2+^ are removed mainly through surface adsorption, ion exchange, and precipitation of sparingly soluble metal carbonates and hydroxides, processes favoured by the increase in pH caused by dolomite dissolution [20]. In the present study, a pronounced pH increase was observed after passage through the filter layer, supporting the potential occurrence of these processes. This mechanism explains the high heavy metal removal observed in the reference column and indicates that the contribution of halloysite to metal retention was secondary to the alkalising effect of dolomite.

The findings have important practical implications. When rain gardens are designed to treat stormwater contaminated with heavy metals, system performance depends not only on the use of specialised sorbents but also on the appropriate selection of the basic filter-layer components. Dolomite provided very high heavy metal retention, whereas the halloysite amendment increased the capacity of the filter bed to remove nutrients. The results therefore indicate that hybrid mineral filter beds, in which individual components perform different but complementary functions, offer the greatest application potential.

The literature data summarised in Table S3 (Supplementary Materials) confirm that dolomite is among the most effective natural materials used for heavy metal removal from water and wastewater. Most studies report the highest affinity for Pb(II), followed by Cu(II), and the lowest affinity for Zn(II), consistent with the observations of the present study [12,20,21,70,71,72,73,74,75,76,77,78,79].

The dominant mechanisms of metal retention are the increase in pH caused by dolomite dissolution, surface adsorption, ion exchange, and precipitation of sparingly soluble metal carbonates and hydroxides [20,21,70,71,72,73,74,75,76,77,78]. For Pb(II), the formation of cerussite (PbCO_3_), which provides permanent lead immobilisation, is particularly important [12,70,71,72]. For Cu(II), both chemical adsorption and ion exchange involving Ca^2+^ and Mg^2+^ ions in the dolomite structure contribute substantially [70,73]. The lower Zn(II) removal efficiency is attributable to the greater mobility of this metal and the lower stability of the surface complexes and precipitated phases that it forms [71,75,77].

Although the halloysite-amended columns showed excellent heavy metal removal, the present findings indicate that this performance should not be attributed solely to halloysite. All experimental columns contained a dolomite layer, which probably played the dominant role in the removal of Cu, Zn, and Pb. Carbonate minerals such as dolomite are known to increase solution pH and promote surface complexation, ion exchange, and precipitation of sparingly soluble metal carbonates and hydroxides. Halloysite should therefore be regarded primarily as a material that improves nutrient retention, whereas dolomite is the principal component responsible for heavy metal immobilisation. The combination of the two materials probably forms a complementary filtration system in which halloysite improves nutrient removal and dolomite provides effective heavy metal retention [12,74,78].

The results obtained confirm these relationships. Despite the relatively low initial concentrations of Cu, Zn, and Pb, near-complete metal removal was achieved in all columns. This finding indicates that, under the tested conditions, the number of available reactive sites in the dolomite layer substantially exceeded the quantity of introduced pollutants. It also indicates that the sorption capacity of dolomite was not exhausted during the experiments.

The literature further indicates that the sorption properties of dolomite can be enhanced by thermal or chemical activation or by composite formation, which increases the specific surface area and the number of active sorption sites [74,76]. In these studies, the improvement was particularly evident for Cu(II) and Zn(II), whereas Pb(II) removal remained very high even for the natural material [69,71,74]. These findings demonstrate the substantial potential for further optimisation of filter materials intended for stormwater treatment.

pH is another important factor controlling process performance. Both the present results and the literature data indicate that increasing pH promotes heavy metal removal by reducing competition from H^+^ ions for active sites and increasing the contribution of precipitation and surface complexation [20,69,73,78]. It should nevertheless be recognised that, at very high pH, part of the observed metal removal may result from chemical precipitation rather than exclusively from adsorption at the dolomite surface.

From a practical perspective, the results indicate that dolomite should be considered not only a structural component of the filter layer but also an active sorbent responsible for heavy metal immobilisation. In combination with halloysite, it forms a complementary filter system in which dolomite primarily retains heavy metals, whereas halloysite improves nutrient removal. This approach can provide high stormwater treatment efficiency using natural mineral materials of relatively low cost.

The present study evaluated halloysite-amended columns over a limited number of simulated rainfall events and therefore represents only the initial stage of column operation. The results consequently do not permit an assessment of long-term sorption capacity, progressive saturation of active sites, regeneration potential, or clogging processes that may develop during prolonged field operation. Future research should include long-term laboratory and field studies with repeated natural rainfall events to evaluate halloysite durability, changes in hydraulic conductivity, regeneration potential, and the service life of halloysite-amended filters under realistic environmental conditions.

The results should be interpreted in the context of the laboratory scale of the study. The column experiments were conducted under controlled conditions and do not fully reproduce the complexity of field-scale rain gardens, in which pollutant removal is affected by vegetation, microbial activity, seasonal temperature variation, rainfall variability, clogging processes, and variations in stormwater pollutant composition. Moreover, the sorption capacity of the investigated filter materials may gradually decrease during operation under actual conditions. Further studies in full-scale rain gardens are therefore required to verify their long-term performance and practical applicability.

The leachates generated in the experiments were strongly alkaline. pH is one of the key factors affecting nutrient availability, microbial activity, and plant growth, with most biological processes operating optimally within a pH range of 5.5–8.8 [80]. Prolonged pH values above 9 may reduce nutrient availability, disrupt microbial activity, and impair rain garden ecosystem functioning [81]. At the same time, some wetland plant species, including *Iris sibirica*, *Lythrum salicaria*, *Acorus tatarinowii*, and *Sparganium erectum*, exhibit high tolerance to alkaline conditions. Elevated pH therefore does not preclude rain garden operation but may influence plant selection [82].

It should be emphasised, however, that the results were obtained from laboratory experiments conducted without vegetation. In full-scale rain gardens, effluent pH may gradually decrease owing to substrate-buffering processes, contact with atmospheric CO_2_, and the activity of plant roots and microorganisms [5]. The pH values obtained in the present study should therefore be regarded as initial values that require confirmation under field conditions.

## 5. Conclusions

The results of the present study indicate that the use of halloysite as an amendment to the filter layer of laboratory-scale rain gardens may improve the removal efficiency of selected contaminants present in stormwater. The results also suggest that material performance depends on the type of contaminant, sorbent grain size, and hydraulic conditions associated with rainfall dynamics.

1. DIN and SRP removal efficiency: The halloysite amendment increased DIN removal to 82–84% (compared with <80% in the reference column) and SRP removal to 85% (particularly after a 7-day antecedent dry period), demonstrating high affinity for ammonium ions, moderate affinity for phosphates, and limited affinity for nitrates.
2. Mechanisms and grain size effect (1–2 mm): Ammonium ion removal may proceed through ion exchange with surface cations of halloysite, while phosphate removal may occur through adsorption at aluminol and silanol groups and electrostatic interactions; the finer fraction (1–2 mm) provided greater external contact surface area per unit bed volume and may have resulted in higher SRP removal efficiency in column C2. The grain size effect was contaminant-dependent and of limited magnitude under the experimental conditions applied.
3. Nitrate nitrogen leaching: N–NO_3_^−^ concentrations increased in all experimental variants, confirming ineffective nitrate removal and indicating nitrate leaching from the filter material.
4. Heavy metal removal (Cu, Zn, Pb): The very high removal of Cu, Zn, and Pb (below limits of quantification) in all column configurations resulted from the dominant role of dolomite aggregate, while the contribution of halloysite was secondary. Halloysite primarily supported nutrient removal, whereas dolomite was mainly responsible for heavy metal immobilisation. Their combined use provides a promising hybrid filter material for rain garden systems.
5. Grain size effect on DIN: In contrast to phosphates, the influence of halloysite grain size on DIN removal was limited, with slightly more favourable results obtained for the coarser fraction (column C3).
6. Rainfall dynamics and contact time: The largest decreases in DIN and SRP concentrations occurred during the first 30 min of rainfall (first flush effect), after which concentrations in the collected samples gradually increased as the contact time between water and the filter material decreased.
7. Mitigation of alkalisation (pH): The use of dolomite alone (reference column C1) led to strong alkalisation of the studied samples (pH 9.45–9.55), whereas the halloysite amendment effectively attenuated this effect, reducing maximum pH values to the range of 8.21–8.95. Hybrid filter beds combining the sorption properties of halloysite with the high effectiveness of dolomite for heavy metal immobilisation therefore offer the greatest application potential and represent a promising approach to rain garden design.

## Figures and Tables

**Table 1:** Structure of the filter columns—composition of individual layers.

Layer	Function	C1	C2	C3
Top (0–5 cm)	Erosion protection; stream dispersion	Gravel (φ = 0.063–20 mm)	Gravel (φ = 0.063–20 mm)	Gravel (φ = 0.063–20 mm)
Middle (5–50 cm)	Bioretention; pollutant removal	Sand (0.2–2 mm)	80% Sand (0.2–2 mm) +20% Halloysite (1–2 mm)	80% Sand (0.2–2 mm) +20% Halloysite (2–4 mm)
Bottom (50–80 cm)	Drainage and retention; water discharge	Dolomite aggregate (2–20 mm)	Dolomite aggregate (2–20 mm)	Dolomite aggregate (2–20 mm)

**Table 2:** Composition of the nutrient rainfall and metal rainfall.

Type	Component	Source	Concentration mg·L^–1^
Nutrient rainfall	N–NH_4_^+^	NH_4_Cl	3.89 ± 0.39
	N–NO_3_^–^	KNO_3_	3.39 ± 0.68
	P–PO_4_^3–^	Na_2_HPO_4_	0.65 ± 0.03
Metal rainfall	Cu	Cu(NO_3_)_2_	2.85 ± 0.43
	Zn	Zn(NO_3_)_2_	5.95 ± 0.89
	Pb	Pb(NO_3_)_2_	1.05 ± 0.16
	N–NO_3_^–^	Pb(NO_3_)_2_, Zn(NO_3_)_2_, Cu(NO_3_)_2_	2.82 ± 0.56

**Table 3:** Stages of the degassing process.

Target Temperature °C	Heating Speed °C·min^−1^	Degassing Time min
80	2	30
120	2	30
300	5	400

**Table 4:** Analytical methods used for the determination of synthetic rainfall composition.

Parameter	Uncertainty	Method/Reference Standard/Modification
Ammonium (NH_4_^+^)	±10%	Spectrophotometry; Visocolor Powder Pillows Ammonium test kit (0.03–0.80 mg·L^–1^); Nanocolor UV/VIS II (Macherey-Nagel, Düren, Germany)
Nitrate (NO_3_^–^)	±20%	Spectrophotometry; Nanocolor Nitrate test kit (0.02–1.0 mg N–NO_3_^−^·L^–1^); Nanocolor UV/VIS II (Macherey-Nagel, Düren, Germany)
Phosphate (PO_4_^3–^)	±5%	Spectrophotometry; Nanocolor Total Phosphorus 15 test kit (0.3–15.0 mg·L^–1^); Nanocolor UV/VIS II (Macherey-Nagel, Düren, Germany)
Copper (Cu), Zinc (Zn), Lead (Pb)	±15%	FAAS; PN-ISO 8288:2002 [40]; flame atomisation (acety-lene/air); Solaar S4 AA (Thermo Fisher Scientific, Waltham, MA, USA); analytical ranges: Cu 0.05–2.00 mg·L^–1^; Zn 0.02–2.00 mg·L^–1^; Pb 0.10–2.00 mg·L^–1^; samples acidified with concentraded HNO_3_ (65%, spectrally pure) at 2 mL·L^–1^; R^2^ > 0.995

**Table 5:** Textural properties of the halloysite sample.

**Specific surface area** **m^2^·g^–1^**	BET surface area (multipoint method)	55.73
	BJH specific surface area	47.05
	DR specific surface area	63.87
**Pore volume** **cm^3^·g^–1^**	Total pore volume, single-point (at p/p_0_ = 0.99)	0.22
	BJH pore volume	0.22
	DR pore volume	0.02
**Pore size** **nm**	Mean pore diameter	15.90
	BJH mean pore diameter	3.06
	DR mean pore diameter	2.02

**Table 6:** Initial and final heavy metal concentrations in Exp. 3 and Exp. 4.

Experiment	Column	Time min.	Cu mg·L^–1^	Zn mg·L^–1^	Pb mg·L^–1^
Exp. 3	C1	0	2.80	6.07	0.96
		30, 60, 90, 120, 150	<0.05	<0.02	<0.10
	C2	0	2.82	6.00	0.95
		30, 60, 90, 120, 150	<0.05	<0.02	<0.10
	C3	0	2.80	5.82	0.92
		30, 60, 90, 120, 150	<0.05	<0.02	<0.10
	C1	0	2.96	5.95	0.99
		30, 60, 90, 120, 150	<0.05	<0.02	<0.10
	C2	0	2.81	5.82	0.96
		30, 60, 90, 120, 150	<0.05	<0.02	<0.10
	C3	0	2.78	5.76	0.93
		30, 60, 90, 120, 150	<0.05	<0.02	<0.10
Exp. 4	C1	0	3.16	6.51	1.08
		5, 10, 15, 20, 25, 30, 45	<0.05	<0.02	<0.10
	C2	0	3.10	6.04	1.08
		5, 10, 15, 20, 25, 30, 45	<0.05	<0.02	<0.10
	C3	0	3.06	5.97	1.07
		5, 10, 15, 20, 25, 30, 45	<0.05	<0.02	<0.10

## Data Availability

The original contributions presented in this study are included in the article/Supplementary Material. Further inquiries can be directed to the corresponding author.

## Supplementary Materials

The following supporting information can be downloaded at: https://www.mdpi.com/article/10.3390/ma19163466/s1, Figure S1: Comparison of mean pH changes in the three columns as a function of time during (a) Exp. 1, (b) Exp. 2, (c) Exp. 3, and (d) Exp. 4.; Table S1: Nutrient removal efficiency from water and wastewater using halloysite: comparison with literature data; Table S2: Heavy metal removal efficiency from water and wastewater using halloysite: comparison with literature data; Table S3: Heavy metal removal efficiency from water and wastewater using dolomite: comparison with literature data.

## Abbreviations

The following abbreviations are used in this manuscript:

BET	Brunauer–Emmett–Teller method
BJH	Barrett–Joyner–Halenda method
C1, C2, C3	designations of columns
DIN	dissolved inorganic nitrogen
DR	Dubinin-Radushkevich method
EDS	Energy Dispersive X-ray Spectroscopy
Exp. 1	Experiment 1 described in this article
Exp. 2	Experiment 2 described in this article
Exp. 3	Experiment 3 described in this article
Exp. 4	Experiment 4 described in this article
FAAS	Flame atomic absorption spectrometric
IUPAC	International Union of Pure and Applied Chemistry
NbS	Nature-based solutions
q_e_	adsorption capacity mg·g^–1^, mmol·kg^–1^
S_BET_	surface areas calculated using the Brunauer–Emmett–Teller BET method
SEM	Scanning Electron Microscope
SRP	soluble reactive phosphorus
