# Supplementary material for: Nutrient Removal by Halloysite-Amended Mineral Matrices and Heavy Metal Retention in Rain Gardens Under Dynamic Hydraulic Flow Conditions
Source: Materials (Basel). 2026 Aug 17;19(16):3466. doi: 10.3390/ma19163466 (PMC13514831; doi:10.3390/ma19163466)
Supplement: Supplementary file 1 [file materials-19-03466-s001.zip › materials-4466727-supplementary-FigS1.pdf]

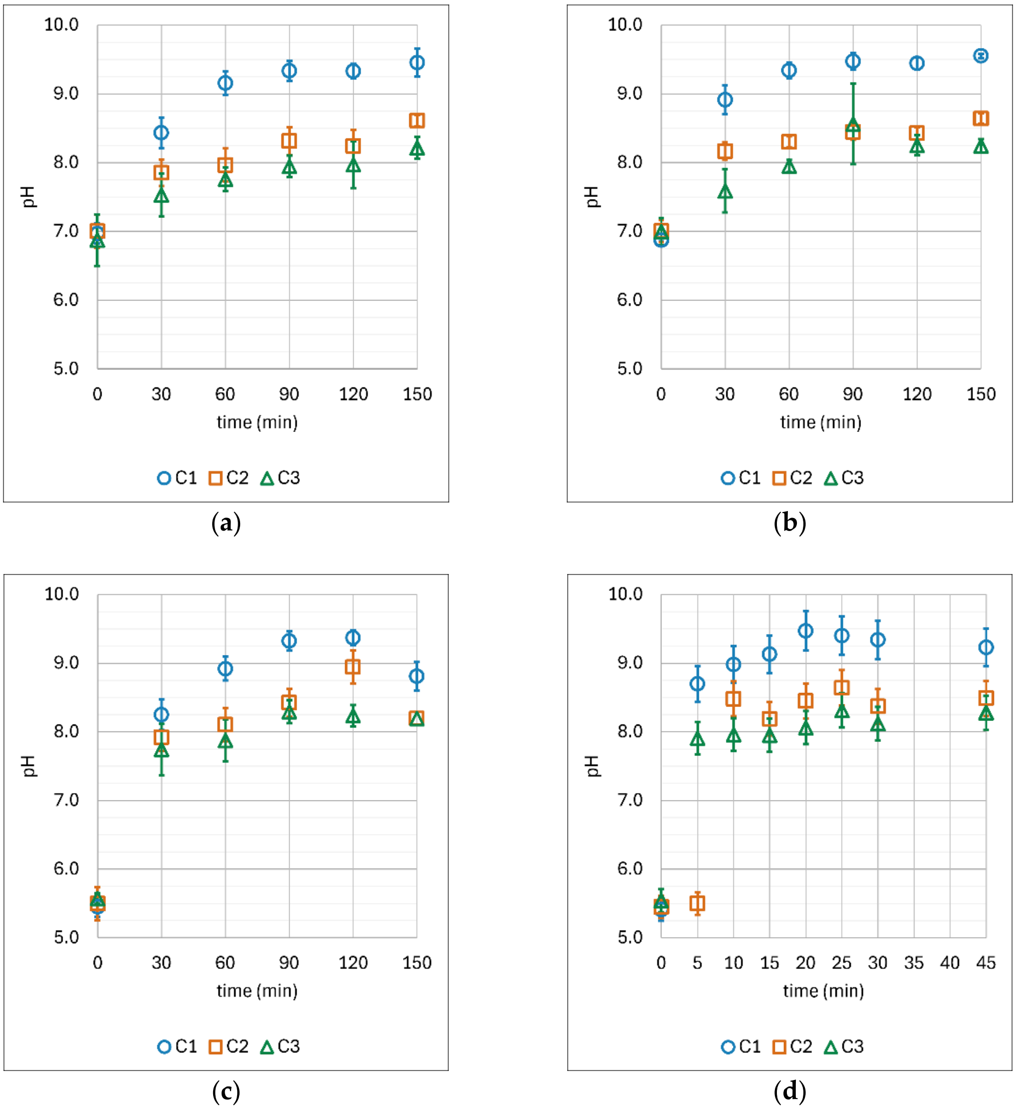

Figure S1. Comparison of mean pH changes in the three columns as a function of time during (a) Exp. 1, (b) Exp. 2, (c) Exp. 3, and (d) Exp. 4.
