# Supplementary material for: Nutrient Removal by Halloysite-Amended Mineral Matrices and Heavy Metal Retention in Rain Gardens Under Dynamic Hydraulic Flow Conditions
Source: Materials (Basel). 2026 Aug 17;19(16):3466. doi: 10.3390/ma19163466 (PMC13514831; doi:10.3390/ma19163466)
Supplement: Supplementary file 1 [file materials-19-03466-s001.zip › materials-4466727-supplementary-TabS1.pdf]

Table S1. Nutrient removal efficiency from water and wastewater using halloysite: comparison with literature data.

Contaminat ion	Material	Initial concentration	Conditions	Removal efficiency / Sorption capacity	Mechanism	Source
NH_4^+	natural halloysite	5.0 mg $NH_4^+ \cdot L^{-1}$	columns; pH = 6.9; halloysite mass C2 = C3 = 1.9 kg; t_c = 7 min; T = 20°C	C2 RE_{mean} = 45%; C3 RE_{mean} = 54%	probably ion exchange; physicochemical adsorption	this study
NH_4^+	weathered halloysite	5–60 mg $NH_4^+ \cdot L^{-1}$ and higher series for isotherms	batch; pH = 4–10; dose 12–72 $g \cdot L^{-1}$; t_c = 45 min	q_{max} = 2.75 mg $NH_4^+ \cdot g^{-1}$;	predominantly NH4+ ↔ Na+, K+, Ca^{2+} ion exchange within halloysite structure; partial surface adsorption	[10]
NH_4^+	zeolite Y from halloysite	10–300 mg $NH_4^+ \cdot L^{-1}$	batch; pH = 3–9; t_c = 30 min	q_e = 38.64 mg $NH_4^+ \cdot g^{-1}$	intensive ion exchange owing to increased surface area and number of active sites after halloysite-to-zeolite transformation	[29]
NH_4^+	chitosan/PAA hydrogel composite with halloysite (30 wt%)	synthetic wastewater; 100–200 mg $N \cdot L^{-1}$	batch; pH = 4–7; equilibrium after ~5 min	q_{max} = 40.9 mg $N \cdot g^{-1}$	NH_4^+ ion exchange; adsorption at hydrogel carboxyl groups; increased active surface area via halloysite micropores	[28]
N–NH_4	natural halloysite	up to 600 mg N–$NH_4 \cdot L^{-1}$	batch; pH = 5.6; T = 288–313 K;	q_{max} = 1.66 $mg \cdot g^{-1}$	NH_4^+ ion exchange for surface cations of halloysite; physicochemical adsorption	[27]
PO_4^{3-}	natural halloysite	2.0 mg PO_4^{3-} $mg \cdot L^{-1}$	columns; pH = 6.9; halloysite mass C2 = C3 = 1.9 kg; t_c = 7 min; T = 20°C	C2 RE_{mean} = 29%, C3 RE_{mean} = 24%	probably adsorption; chemisorption; PO_4^{3-} complexation at Al–OH groups	this study
PO_4^{3-}	halloysite nanotubes (HNTs)	1–100 mg $PO_4^{3-} \cdot L^{-1}$	batch; pH = 7; dawka 40 $g \cdot L^{-1}$; t_c = 24 h	q_{max} = 0.55 mg $P \cdot g^{-1}$	electrostatic adsorption and phosphate complexation at Al–OH groups	[31]
PO_4^{3-}	Fe oxide-modified halloysite	0.5–100 mg $PO_4^{3-} \cdot L^{-1}$	batch; pH = 2–10;	q_e = 5.46 mg $P \cdot g^{-1}$	formation of Fe–PO_4 complexes;	[30]

			t_c = 4 h		chemisorption and ligand adsorption	
$P–PO_4^{3-}$	natural halloysite nanotubes (HNTs), two forms: cylindrical and prismatic	2.5–8.0 mg $P–PO_4^{3-}$·L^{-1}	batch; pH = 3–11	q_{max} = 1.3 mg·g^{-1} (cylindrical), q_{max} = 0.5 mg·g^{-1} (prismatic)	adsorption mainly at Al–OH; edges; higher capacity associated with larger specific surface area and nanotube size (lumen)	[13]
NO_3^-	natural halloysite	15.0 mg NO_3^-·L^{-1}	columns; pH = 6.9; halloysite mass C2 = C3 = 1.9 kg; t_c = 7 min; T = 20°C	C2 RE_{mean} = –17% C3 RE_{mean} = –7%	probably adsorption	this study
$N–NO_3^-$	CTAB-modified halloysite nanotubes (HNTs–CTAB)	optimal: 100 mg NO_3^-·L^{-1}; isotherms: 10–120 mg NO_3^-·L^{-1},	pH = 4; T = 25°C; 40 mg adsorbent per 10 ml solution; t_c = 3h	q_{max} = 47.4 mg·g^{-1}	electrostatic adsorption of NO_3^- anions on positively charged halloysite surface after CTAB modification	[51]
NO_3^- in solution as anions accompanying heavy metal cations	halloysite with triethanolamine (TEA); halloysite with diethanolamine (DEA)	0.005–5 mmol NO_3^-·L^{-1}	batch, pH = 2–6.5; t_c = 24 h	q_{max} = 29 mmol NO_3^-·kg^{-1} (TEA); q_{max} = 4 mmol NO_3^-·kg^{-1} (DEA)	Removal strongly pH-dependent; q_{max} at pH 2–3.5; above pH = 5, nitrate binding capacity diminished; increase in amino and hydroxyl groups may enhance sorption of anions and cations	[18]

Abbreviations

The following abbreviations are used in this manuscript:

q_e	adsorption capacity mg·g^{-1}, mmol·kg^{-1}
q_{max}	maximum adsorption capacity mg·g^{-1}, mmol·kg^{-1}
RE_{mean}	mean removal efficiency %
t_c	contact time
