# Supplementary material for: Nutrient Removal by Halloysite-Amended Mineral Matrices and Heavy Metal Retention in Rain Gardens Under Dynamic Hydraulic Flow Conditions
Source: Materials (Basel). 2026 Aug 17;19(16):3466. doi: 10.3390/ma19163466 (PMC13514831; doi:10.3390/ma19163466)
Supplement: Supplementary file 1 [file materials-19-03466-s001.zip › materials-4466727-supplementary-TabS2.pdf]

Table S2. Heavy metal removal efficiency from water and wastewater using halloysite: comparison with literature data.

Contaminat ion	Material	Initial concentration	Conditions	Removal efficiency / Sorption capacity	Mechanism	Source
Cu(II)	natural halloysite	40 mg Cu·L^{-1}	batch; 0.8 g·50 mL^{-1}; pH = 6.2; t_c = 80 min; T = 25°C	RE = 85.2%; q_e = 2.14 mg·g^{-1}	electrostatic attraction (pH > pH_{PZC}); competition with other ions	[65]
Cu(II)	halloysite modified with APTES and succinic acid	10–80 mg Cu·L^{-1}	batch; pH = 2–6;	data not available	chelation of Cu^{2+} by amino and carboxyl groups on modified halloysite surface	[15]
Zn(II)	natural halloysite	40 mg Zn·L^{-1}	batch; 0.7 g·50 mL^{-1}; pH = 5.6; t_c = 100 min; T = 30°C	RE = 67.09%	electrostatic adsorption and partial ion exchange	[67]
Zn(II)	natural halloysite (H), calcined (HC), acid-activated (HA)	0.005–5.0 mmol Zn·L^{-1} (H); 0.05–50.00 mmol Zn·L^{-1} (HC/HA)	batch; adsorption selectivity studies; pH = 5; t_c = 24 h; comparison of different halloysite forms	q_e = 2.3 mmol·kg^{-1} (H); q_e = 203.2 mmol·kg^{-1} (HC); no adsorption (HA)	ion exchange; surface complexation	[68]
Pb(II)	natural halloysite	40 mg Pb·L^{-1}	batch; 0.7 g·50 mL^{-1}; pH = 5.31; t_c = 80 min; T = 30°C;	RE = 90.75%	strong inner-sphere adsorption of Pb^{2+} at ≡Al–OH and ≡Si–OH groups	[67]
Pb(II)	natural and chemically activated halloysite	10 mg Pb·L^{-1}	batch; pH = 3.0–3.5 and 6.0–6.5; BET, DFT, and SEM analysis	<LOQ	adsorption in interlayer spaces; migration of ions into the halloysite structure	[16]
Pb(II)	halloysite/alginate composite	10–1000 mg Pb·L^{-1}	batch; Langmuir analysis; pseudo-second-order kinetics	q_e = 325 mg·g^{-1}	$Pb^{2+} \leftrightarrow Ca^{2+}$ ion exchange and physisorption within halloysite nanotubes	[17]

Pb(II)	natural halloysite (H), calcined (HC), acid-activated (HA)	0.005–5.0 mmol Pb·L^{-1} (H);<br>0.05–50.00 mmol Pb·L^{-1} (HC/HA)	batch;<br>pH=5;<br>t_c = 24 h;<br>comparison of different halloysite forms	q_e = 35.9 mmol·kg^{-1} (H);<br>q_e = 207 mmol·kg^{-1} (HC);<br>q_e = 207.3 mmol·kg^{-1} (HA)	ion exchange; surface complexation	[68]
Cu(II), Pb(II), Zn(II)	kaolinite minerals	50–300 mg·L^{-1}	batch;<br>pH = 5.0±0.1;<br>t_c = 6 h;<br>T = 298 K;	adsorption:<br>Pb > Cu > Zn	surface complex formation and electrostatic interactions	[14]
Cu(II), Zn(II)	halloysite nanotube derivatives (HNT derivatives)	25–400 mg·L^{-1}	batch;<br>effect of time, temperature, and pH	adsorption:<br>Cu^{2+}>Zn^{2+}	chemisorption and ion exchange; involvement of hydroxyl groups	[66]

Abbreviations

The following abbreviations are used in this manuscript:

APTES	3-aminopropyltriethoxysilane
BET	Brunauer–Emmett–Teller method
BJH	Barrett–Joyner–Halenda method
DFT	Density Functional Theory
q_e	adsorption capacity mg·g^{-1}, mmol·kg^{-1}
LOQ	Limit of Quantification
RE	removal efficiency %
S_{BET}	surface areas calculated using the Brunauer–Emmett–Teller BET method
SEM	Scanning Electron Microscope
t_c	contact time
