# Supplementary material for: Nutrient Removal by Halloysite-Amended Mineral Matrices and Heavy Metal Retention in Rain Gardens Under Dynamic Hydraulic Flow Conditions
Source: Materials (Basel). 2026 Aug 17;19(16):3466. doi: 10.3390/ma19163466 (PMC13514831; doi:10.3390/ma19163466)
Supplement: Supplementary file 1 [file materials-19-03466-s001.zip › materials-4466727-supplementary-TabS3.pdf]

Table S3. Heavy metal removal efficiency from water and wastewater using dolomite: comparison with literature data.

Contamination	Material	Initial concentration	Conditions	Removal efficiency / Sorption capacity	Mechanism	Source
Cu(II)	natural dolomite	3 $mg \cdot L^{-1}$	columns with 30 cm dolomite layer (mass 7.4 kg); pH = 5.4, t_c = 7 min; T = 20°C	<LOQ	probably alkalisation, precipitation, and sorption	this study
Cu(II)	natural powdered dolomite	~127–1016 $mg \cdot L^{-1}$	batch; pH = 5; 0.2 g sorbent; t_c = 60 min	RE >85%; q_{max} = 8.26 $mg \cdot g^{-1}$;	Ca^{2+}/Mg^{2+} ion exchange and surface complexation	[69]
Cu(II)	natural dolomite	10–60 mg $Cu \cdot L^{-1}$	pH = 2–8; t_c = 5 min–6 h; T = 20–50°C;	data not available	chemical adsorption and surface complexation	[72]
Cu(II)	natural dolomite	50–150 mg $Cu \cdot L^{-1}$	adsorption–desorption system	data not available	adsorption at carbonate and hydroxyl groups	[72]
Cu	natural dolomite	10–60 mg $Cu \cdot L^{-1}$	pH = 2–8; t_c = 120 min; T = 20–50°C;	q_{max} = 0.475 $mg \cdot g^{-1}$	adsorption dependent on concentration, temperature, and pH	[73]
Zn(II)	natural dolomite	6 $mg \cdot L^{-1}$	column with 30 cm dolomite layer (mass 7 kg); pH = 5.4,	<LOQ	probably alkalisation, precipitation, and sorption	this study
Zn(II)	waste dolomite dust	500–2000 mg $Zn \cdot L^{-1}$	batch; sorbent mass = 0.5–2 g; t_c = 45–180 min	max. 47% removal at 500 $mg \cdot L^{-1}$	ion exchange and electrostatic adsorption	[70]
Zn(II)	phosphate-modified dolomite	5 mg $Zn \cdot L^{-1}$	dynamic flow; T = 25°C	RE = 90–95% Zn^{2+} removal	formation of zinc phosphates and surface adsorption	[78]
Zn(II)	natural sorbent containing dolomite	laboratory Zn^{2+} solutions	pH = 6.5; kinetic and isotherm studies	q_e = 2.860 $mmol \cdot g^{-1}$ (~187 $mg \cdot g^{-1}$)	Langmuir adsorption; ion exchange	[74]
Zn(II)	thermally activated dolomite	neutral metal-bearing drainage	flow-through system	data not available	increased CaO/MgO reactivity	[75]
Zn	half-burnt dolomite (thermal dissociation of natural dolomite at 650–750°C)	5 $mg \cdot L^{-1}$	laboratory scale; glass columns 2 cm diameter; road runoff	q_e = 3420 $mg \cdot kg^{-1}$	precipitation of zinc as hydroxides and carbonates resulting from high leachate pH (~10)	[77]

Zn	dolomite as component of filter mixtures	100–5000 μg·L^{-1}	plexiglass columns, 32 mm and 100 mm diameter; road and roof runoff	RE = 94.5%; q_e = 4420 mg·kg^{-1}	higher pH promotes metal precipitation as oxides and carbonates	[12]
Zn	raw dolomite (RD); calcined dolomite (CD) (firing at 700°C for 2 h)	365±18 mg·L^{-1}	batch; 15 g solid sample; 150 mL deionised water; t_c = 6 h;	q_e = 1 mg·L^{-1} (RD); q_e < 1 mg·L^{-1} (CD)	surface adsorption on RD and coprecipitation with iron (oxy)hydroxides; precipitation of $Zn(OH)_2$ reducing zinc solubility (CD)	[76]
Pb(II)	natural dolomite	1 mg·L^{-1}	column with 30 cm dolomite layer (mass 7 kg); pH = 5.4,	<LOQ	probably alkalisation, precipitation, and sorption	this study
Pb(II)	natural powdered dolomite	414–3310 mg·L^{-1}	batch; pH = 5; t_c = 60 min	q_{max} = 21.74 mg·g^{-1}; RE >85%	$PbCO_3$ precipitation and surface complexation	[69]
Pb(II)	waste dolomite dust	500–2000 mg Pb·L^{-1}	batch; t_c = 45–180 min	RE = ~100%	$PbCO_3$ precipitation and surface complexation	[70]
Pb(II)	natural dolomite	1–50 mg Pb·L^{-1}	t_c up to 15 days;	data not available	adsorption and cerussite phase nucleation	[71]

Abbreviations

The following abbreviations are used in this manuscript:

q_e	adsorption capacity mg·g^{-1}, mmol·kg^{-1}
q_{max}	maximum adsorption capacity mg·g^{-1}, mmol·kg^{-1}
LOQ	Limit of Quantification
RE	removal efficiency %
t_c	contact time
